# Transcriptome Analysis of Orange Head Chinese Cabbage (*Brassica rapa* L. ssp. *pekinensis*) and Molecular Marker Development

**DOI:** 10.1155/2017/6835810

**Published:** 2017-04-02

**Authors:** Jingjuan Li, Yihui Zhang, Qian Ding, Huayin Li, Lifeng Liu, Fengde Wang, Jianwei Gao

**Affiliations:** Institute of Vegetables and Flowers, Shandong Academy of Agricultural Sciences and Shandong Key Laboratory of Greenhouse Vegetable Biology and Shandong Branch of National Vegetable Improvement Center, Jinan 250100, China

## Abstract

Due to the visual appearance and high carotenoid content, orange inner leaves are a desirable trait for the Chinese cabbage. To understand the molecular mechanism underlying the formation of orange inner leaves, the *BrCRTISO* (Bra031539) gene, as the *Br-or* candidate gene, was analyzed among the white and orange varieties, and 7 single nucleotide polymorphisms (SNPs) were identified. However, only one SNP (C^952^ to T^952^) altered the amino acid sequence, resulting in a mutation from Leu^318^ to Phe^318^ in the orange varieties. Additionally, we analyzed differentially expressed genes (DEGs) between the orange and white F_2_ individuals (14-401 × 14-490) and found four downregulated genes were involved in the carotenoid biosynthesis pathway, which may lead to the accumulation of prolycopene and other carotenoid pigments in the orange inner leaves. In addition, we developed a novel InDel marker in the first intron, which cosegregates with the phenotypes of orange color inner leaves. In conclusion, these findings enhance our understanding of the underlying mechanism of pigment accumulation in the inner leaves of the Chinese cabbage. Additionally, the SNP (C^952^ to T^952^) and the InDel marker will facilitate the marker-assisted selection during Chinese cabbage breeding.

## 1. Introduction

Carotenoids, as a group of colorful pigments, are responsible for the yellow, orange, or red color of fruits and flowers [1]. These pigments play an essential role in plant growth and development, such as harvesting lighting energy and preventing photo-oxidation during photosynthesis [2, 3]. Plant carotenoids also play important roles in human nutrition and health, either functioning as precursors for vitamin A synthesis or offering protection to consumers against cardiovascular diseases, cancer, and age-related eye diseases [4–8].

Chinese cabbage (*Brassica rapa* L. ssp. *pekinensis*) is an important vegetable crop planted worldwide, especially in Asia. The color of the inner head leaves is usually white, yellow, or orange. The orange color of the inner leaves is a result of the accumulation of prolycopene and other carotenoid pigments [9–11]. In recent years, the orange head Chinese cabbage varieties have gradually captured the attention of breeders and consumers, since they have an attractive color and higher carotenoids compared with the white and yellow head cabbage.

Previous studies have shown that the accumulation of carotenoids in the inner leaves of Chinese cabbage is controlled by a single recessive gene, *Br-or* (Feng et al. [12]; Matsumoto et al. [13]; Zhang et al. [14]). In recent years, the *BrCRTISO* (Bra031539) gene has been identified as the *Br-or* candidate by several research groups. Many insertions/deletions were identified in this gene [9–11, 15], resulting in mutant BrCRTISO proteins, which cannot catalyze the conversion from prolycopene to all-*trans*-lycopene [15]. Furthermore, studies have also shown mutations of the *BrCRTISO* gene dramatically alter the expressions of many other genes [11, 15]. However, controversial results were reported. For example, Su et al. [15] found the enzymes are upregulated in the upstream pathway of the all-*trans*-lycopene biosynthesis and downregulated in the downstream pathway in the orange inner leaves using a RT-qPCR method, while Zhang et al. [11] did not observe any expression differentiations of these genes using a RNA-seq technology. In the present study, we employed a high-throughput sequencing method to identify the differentially expressed genes (DEGs) between the orange and white F_2_ individuals. This study will provide new insights into the underlying mechanism of the pigment accumulation in the inner leaves of the Chinese cabbage. In addition, we analyzed the promoter regions and the coding sequence of the *BrCRTISO* gene in the white and orange head Chinese cabbage varieties and developed one SNP and one InDel marker, which cosegregate with the orange color phenotype of the inner leaves. These markers will provide a valuable tool in the process of breeding of orange-colored cultivars.

## 2. Materials and Methods

### 2.1. Plant Materials

In this study, a total of 25 cultivars were used. Cultivars with white or yellow inner leaves include 14-401, 663, 1466, 1469, 1492, 1505, 1510, 1720, Hanxiu, Jindianchunwang, Kaichun, and Ribenxiayang. Cultivars with orange inner leaves include 14-490, 1480, 14-102, 14-245, 14-253, 14-257, 14-277, 14-426, 14-662, 14-669, Changyanjubao, Shenmengjuhongxin, and Shenshijuhongxin. The F_2_ individuals (14-401 × 14-490) were grown in the farm station at the Vegetable and Flower Research Institute, Shandong Academy of Agricultural Sciences, Jinan, Shandong Province, China. The inner leaves were harvested and stored in liquid nitrogen for DNA and RNA extraction.

### 2.2. cDNA and gDNA Analysis for the *BrCRTISO* Gene

Total RNA was isolated from Chinese cabbage leaves using the TRNzol-A^+^ reagent (Tiangen, Beijing, China) and treated with RNase-free DNase I (Takara, Dalian, China) for 45 min according to the manufacturer's protocol. First-strand cDNA was synthesized from 1 *μ*g of total RNA using a PrimeScript 1st Strand cDNA Synthesis Kit (Takara). The genome DNA was extracted from the inner leaf tissues using a DNA quick Plant System (Tiangen). The *BrCRTISO* gene sequence was divided into two parts (part I and part II) for PCR amplification separately. Two specific primers, Bra031539-G1 and Bra031539-G2 (see Table S1 in Supplementary Material available online at https://doi.org/10.1155/2017/6835810), were designed for amplification of part I and part II, respectively. By using the Bra031539-G2 primer, we were unable to amplify part II sequence of the *BrCRTISO* gene in the 14-490 cultivar. Instead, a genome walking kit (Takara) was used to amplify this sequence. The primers (Supplementary Material: Table S1), including Bror-walking-1, Bror-walking-2, and Bror-walking-3, were designed based on part I sequence. Additionally, the promoter region of the *BrCRTISO* gene was also cloned using the primer Bra031539-P (Supplementary Material: Table S1). PCR reaction was performed in a 50 *μ*l reaction system containing 20 ng template DNA/cDNA, 5.0 *μ*l 10 × LA PCR buffer, 3.0 *μ*l 2.5 mM dNTPs, 2 U LA Taq polymerase (Takara), and 1 *μ*l 10 *μ*M of primers. The amplified products were inserted into the pMD18-T vector (Takara) and sequenced using the Sanger method on an ABI3730XL sequence platform (Applied Biosystems, Foster City, CA).

### 2.3. Validation of the SNP (C^952^ to T^952^) in Different White and Orange Cultivars and F_2_ Populations

To validate the SNP (C^952^ to T^952^), the DNA sequence that contains this site was PCR amplified in different white and orange cultivars and F_2_ populations using the specific primer (OR-SNP^952^, Supplementary Material: Table S1). PCR reaction was performed in a 50 *μ*l reaction system containing 20 ng template DNA, 5.0 *μ*l 10 × LA PCR buffer, 3.0 *μ*l 2.5 mM dNTPs, 2 U LA Taq polymerase (Takara), and 1 *μ*l 10 *μ*M of primers. The amplified products were inserted into the pMD18-T vector (Takara) and sequenced using the Sanger method on an ABI3730XL sequence platform (Applied Biosystems).

### 2.4. cDNA Library Preparation and Sequencing

For high-throughput sequencing, the total RNA was extracted from the inner leaves of the F_2_ individuals using a TRNzol-A^+^ reagent (Tiangen). The same amount of total RNA from 20 F_2_ individuals with white or orange inner leaves was pooled together. The mRNA was purified from the pooled total RNA using Sera-mag Magnetic Oligo (dT) Beads (Illumina Inc., San Diego, CA, USA) and fragmented by adding the fragmentation buffer. With these fragmented mRNAs (about 200 bp) as templates, we synthesized the first-strand cDNAs using a random hexamer primer and the M-MuLV reverse transcriptase (Invitrogen, San Diego, CA). The second-strand cDNA synthesis was subsequently performed, and RNase H was added to remove the RNAs. After adenylated at the 3′-end, the double-stranded cDNA was ligated to the sequencing adapters. Finally, the ligated fragments of ~300 bp were excised and enriched by PCR for 18 cycles. Library quality was assessed using an Agilent 2100 Bioanalyzer system (Agilent Technologies Inc., CA, USA). Libraries were sequenced on an Illumina HiSeq™ 2000 platform (Illumina Inc., San Diego, CA, USA).

### 2.5. Data Analysis

The adapters, empty reads, and low-quality reads (reads in which unknown bases are more than 5% or more than half of their bases have a quality score of less than 5) were removed from the raw reads. The clean reads were aligned to the *B*. *rapa* (Chiifu-401) reference genome (http://brassicadb.org/brad/) using the SOAP2 software with default parameters [16]. After filtering out the reads mapped to multiple reference genes, we obtained a list of unambiguous clean reads. The total reads that were fully mapped to exons were counted, and the expression levels for each gene were calculated.

### 2.6. Differentially Expressed Genes

To identify the DEGs, the gene expression levels were first calculated by the reads per kb per million reads (RPKM) method [17]. Then, the genes were screened using a protocol developed by Audic and Claverie [18]. A threshold, combining FDR (false discovery rate) ≤ 0.001 and an absolute value of log_2_ ratio ≥ 1, was used for the identification of DEGs.

### 2.7. RT-qPCR Analysis

To validate the RNA-seq data, ten potential DEGs were randomly selected and analyzed by the real-time quantitative PCR (RT-qPCR) using the SYBR Green PCR Master Mix (Takara) and the IQ5 RT-qPCR system (Bio-Rad, Hercules, CA, USA). The expression patterns of the *BrCRTISO* (Bra031539) gene were also detected using the RT-qPCR method in the F_2_ individuals with the white or orange inner leaves. For RT-qPCR analysis, the total RNA isolation and first-strand cDNA synthesis were performed as described above. The primers are listed in Supplementary Material Table S1. The *actin* gene was used as a reference for data normalization. RT-qPCR conditions were as follows: 94°C for 2 min, followed by 45 cycles of reaction (94°C for 20 s, followed by 60°C for 30 s). The relative expression levels were calculated from three replicates using the comparative Ct (threshold cycle) method after normalization to an *actin* gene as a control, and the significance was determined with the SPSS software (SPSS 17.0, IBM, Chicago, IL, USA) (*p* < 0.05).

### 2.8. Development of DNA Molecular Markers

A specific primer (Bror-intron 1, Supplementary Material: Table S1) was designed based on the genetic variations found in the *BrCRTISO* intron 1 sequence from the 14-401 and 14-490 varieties. The genotypes of 25 Chinese cabbage breeding lines and cultivars with different inner leaf colors were detected using this primer. PCRs were performed in a 25 *μ*l reaction system containing 0.2 *μ*M of primers, 12.5 *μ*l 2 × Taq PCR Master Mix, and 10 ng gDNA. The amplified products were separated on an 8% denaturing polyacrylamide gel.

## 3. Results and Discussion

### 3.1. Sequence Analysis of the *BrCRTISO* Gene in the White and Orange Head Chinese Cabbages

**Figure 1:**
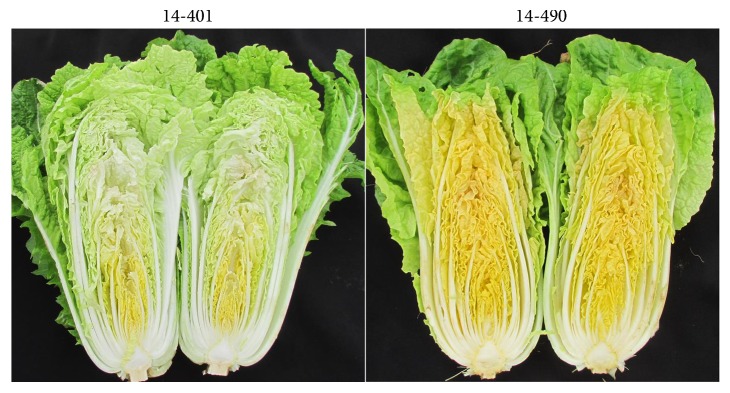
The phenotypes of the 14-401 and 14-490 varieties. 14-401 has the white inner leaves, and 14-490 has the orange inner leaves.

In recent years, several groups have found that the *BrCRTISO* gene is a *Br-or* candidate gene by the genetic mapping method [12–14]. However, the *BrCRTISO* gene in different varieties with orange inner leaves exhibits significant difference in cDNA and genomic sequences [9, 10, 15]. For example, a 501 bp insertion was found at the 3′-end in the *BrCRTISO* gene of the 12-9 cultivar [15], while there is no such an insertion in the A21530 cultivar [10]. In this study, we analyzed the genomic and cDNA sequences of *BrCRTISO* in the 14-401 and 14-490 cultivars with white and orange inner leaves (Figure 1), respectively. By comparing to the genomic sequence of *BrCRTISO* from the Chiifu-401 cultivar (Brassica database, http://brassicadb.org/brad/), we identified many variations, including SNPs and InDels in the 14-490 cultivar, most of which were mapped to introns. Especially in the first intron, 5 DNA insertions and 38 SNPs were detected. For exons, the prominent variations were found in the last exon, where the whole exon was replaced by a large unknown DNA fragment (Supplementary Material: File S1). In the first exon, one deletion and 41 SNPs were detected. Additionally, we found that the genomic sequence of *BrCRTISO* in the 14-490 and 12-9 cultivars were the same.

**Figure 2:**
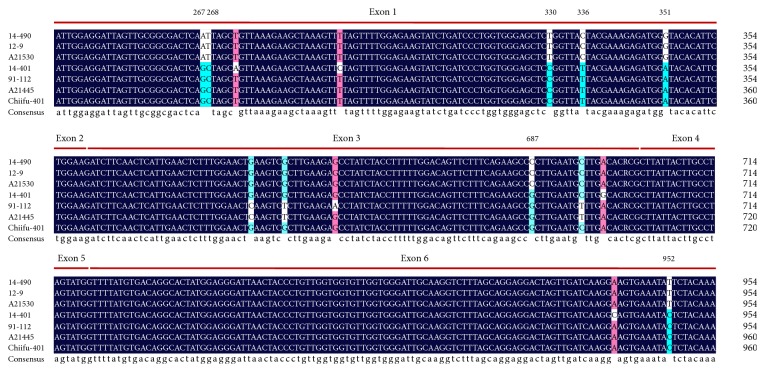
Polymorphic sequences of the *BrCRTISO* genes in the white and orange Chinese cabbage varieties. 14-401, 91-112 [15], A21445 [10], and Chiifu-401 (from Brassica database, BRAD) are the cabbages with the white inner leaves. 14-490, 12-9 [15]), and A21530 [10] are the cabbages with the orange inner leaves. The red lines represent the exons.

In a previous study, 53 SNPs and one InDel were found in the ORF sequence of the *BrCRTISO* gene between an orange head cabbage (A21530) and a white head cabbage (A21445). These variations lead to two glutamic acid (E) deletions and 12 amino acid mutations in A21530, resulting in an inactivated BrCRTISO protein [10]. Comparing the ORF sequence of the *BrCRTISO* genes between an orange head cabbage cultivar (12-9) and a white cultivar (91-112), Su et al. [15] found a number of SNPs, which lead to two mutations (S^19^ to F^19^ and F^316^ to L^316^), and a 501 bp insertion at the nucleotide position 1,692nd in the 12-9 cultivar. These variations also lead to the inactivation of the BrCRTISO protein. In this study, by comparing the ORF sequence of the *BrCRTISO* genes between the orange head cabbages (14-490, 12-9, and A21530) and the white head cabbages (14-401, A21445, 112-91, and Chiifu-401), we found 7 SNPs that were related to the color variation of the inner leaves (Figure 2; Supplementary Material: File S2). However, only one SNP (C^952^ to T^952^) resulted in a mutation in amino acid sequence (L^318^ to F^318^). Additionally, this SNP (C^952^ to T^952^) was cosegregated with the *Br-or* locus (Supplementary Material: Figure S1). Thus, this SNP can be used for marker-assisted selection in the orange head Chinese cabbage.

### 3.2. Differentially Expressed Genes between the Orange Head and White Head F_2_ Individuals

In previous studies, accumulation of carotenoid pigments was detected in the orange inner leaves, including prolycopene, lutein, proneurosporene, pro-*ξ*-carotene, *ξ*-carotene, 9-*cis*-b-carotene, *β*-carotene, and neurosporene [9–11, 15]. The accumulation of the carotenoid pigments is due to the expression changes of related genes caused by the inactivation of the BrCRTISO protein. To identify the DEGs between the orange and white F_2_ individuals (14-401 × 14-490), high-throughput RNA-seq analyses were carried out from the libraries for the orange and white inner leaves. After filtering out the low-complexity reads, the low-quality reads, and the repetitive reads, a total of 11,692,280 and 12,426,002 usable reads for the orange and white inner leaves were obtained (Table 1). Furthermore, more than 80% of the clean reads were successfully aligned to the *B*. *rapa* (Chiifu-401) reference genome (http://brassicadb.org/brad/). By statistical analyses, a total of 60 DEGs were identified, including 11 upregulated genes and 49 downregulated genes (Supplementary Material: Table S2). To validate the RNA-seq results, 10 DEGs were randomly selected and analyzed using the RT-qPCR technique. The results showed that the RT-qPCR expression profiles of nine genes were in complete agreement with the RNA-seq data (Table 2), confirming that the RNA-seq profiling was suitable for the quantitative assessment of the DEGs.

According to the annotation results against the NCBI nonredundant (Nr) database using the BLASTx algorithm [19, 20] with a cutoff E-value of 10^−5^, many putative proteins were identified, such as WRKY DNA-binding protein 1 (Bra023983), cytochrome P450 (Bra010598), expansin-like A2 (Bra033563), 3-ketoacyl-CoA synthase 12 (Bra035683), PLAT/LH2 family protein (Bra030871), pathogenesis-related thaumatin-like protein (Bra015659), and alpha-dioxygenase 1 (Bra039120). In addition, we found 24 unannotated proteins in the DEGs. This result is similar to a previous study, in which the authors identified 372 DEGs, including the genes associated with RNA, protein metabolism, process, cell, wall, signaling, and stress response [11]. Our findings suggest that the mutation of the *BrCRTISO* gene has great impact on transcription regulation. Furthermore, the KEGG pathway analysis was performed using the KEGG database (http://www.kegg.jp/kegg/pathway.html). A total of 14 DEGs were found in five significantly enriched KEGG pathways (*Q* value ≤ 0.05), including biosynthesis of secondary metabolites (ko01110), carotenoid biosynthesis (ko00906), stilbenoid, diarylheptanoid and gingerol biosynthesis (ko00945), ubiquinone and other terpenoid-quinone biosynthesis (ko00130), and limonene and pinene degradation (ko00903) (Table 3).

**Figure 3:**
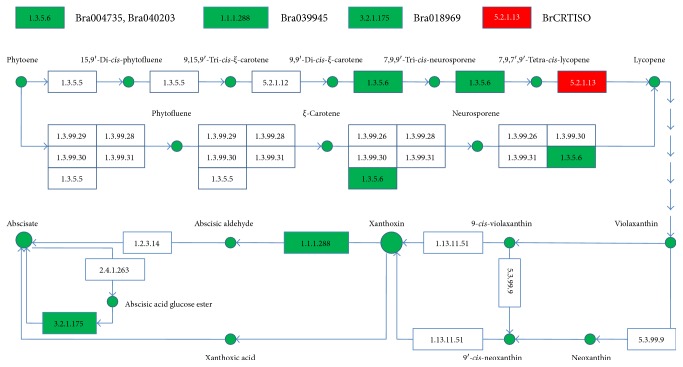
Diagram showing the relationship between the downregulated DEGs and the carotenoid biosynthesis pathway (ko00906, http://www.kegg.jp/kegg-bin/show_pathway?ko00906). The green boxes represent the downregulated genes in the orange inner leaves, and the red box represents the mutated *BrCRTISO* gene.

Several studies have suggested that the accumulation of the carotenoid pigments contributes to the color change of the inner leaves in the Chinese cabbage [9–11]. Carotenoid biosynthesis is a complex process, involving a cascade of pathways (ko00906, http://www.kegg.jp/kegg-bin/show_pathway?ko00906). Four DEGs, including Bra004735, Bra018969, Bra039945, and Bra040203, were found to be involved in the carotenoid biosynthesis. Among these four DEGs, Bra004735 and Bra040203 were annotated as a GDSL-motif lipase/hydrolase family protein and a Li-tolerant lipase 1, respectively, which are involved in catalyzing the conversion of 9,9′-di-*cis*-*ξ*-carotene to 7,9,9′-tri-*cis*-neurosporene, *ξ*-carotene to neurosporene, 7,9,9′-tri-*cis*-neurosporene to 7,9,7′,9′-tetra-*cis*-lycopene, and neurosporene to lycopene (Figure 3). Bra039945 was annotated as a short-chain dehydrogenase/reductase (SDR) family protein, which is involved in catalyzing the conversion of xanthoxin to abscisic aldehyde. Bra018969 was annotated as a beta-glucosidase 1, which is involved in catalyzing abscisic acid glucose ester to abscisate (Figure 3). The Bra004735 and Bra040203 genes are located at the upstream of the *BrCRTISO* gene in the pathway of carotenoid biosynthesis, and their expressions were downregulated in the orange inner leaves (Figure 3), which is probably due to the increased prolycopene contents followed by the inactivation of the BrCRTISO protein. Additionally, the Bra039945 and Bra018969 genes were also downregulated in the orange inner leaves. The inactivation of the BrCRTISO protein and the decreased activities of these four factors may increase the accumulation of other pigments, like *ξ*-Carotene, proneurosporene, and neurosporene in orange inner leaves [9–11]. Controversial results have been reported by Zhang et al. [11] and Su et al. [15]. For example, Su et al. [15] found the enzymes are upregulated in the upstream pathway of all-*trans*-lycopene biosynthesis, and the enzymes are downregulated in the downstream pathway of all-*trans*-lycopene biosynthesis in the orange inner leaves. However, Zhang et al. [11] found these carotenoid biosynthesis genes are not differentially expressed. Gene regulation involves a complicated network of events, which can be regulated by many factors. By using the white and orange F_2_ individuals in our study, the false positive rate was likely to be greatly reduced.

The expression level of the *BrCRTISO* gene was not significantly different between the orange and white F_2_ individuals according to our RNA-seq and RT-qPCR results (Table 2). This result is inconsistent with some previous studies which show that the mRNA level of a *Br-or* candidate gene (Bra031539) is lower in the orange inner leaves than in the white inner leaves [10, 15]. To understand the difference, the promoter sequence of Bra031539 was analyzed. We amplified about 1.3 kb upstream sequence of the *BrCRTISO* genes in 14-401, 14-490, and many other cultivars (Supplementary Material: File S3). One significant finding was a 90 bp DNA deletion in the gene-promoter region, 471 bp upstream of the *BrCRTISO* gene in 14-490. Additionally, the promoter sequences for the *BrCRTISO* genes were compared (14-401 versus 91-112 and 14-490 versus 12-9). Many SNPs and InDels were found between 14-401 and 91-112, while the genes had no difference between 14-490 and 12-9 (Supplementary Material: File S3), which explains the different results obtained by our study and others. Similar to our results, Lee et al. [9] showed there is no significant difference for the expression level of the Bra031539 mRNA between the orange head and white head cultivars.

### 3.3. Development of DNA Molecular Markers for Detection of Cabbage Genotypes

Several markers have been developed based on the sequence differences between the white and orange Chinese cabbage varieties [9–11]. But these markers cannot be commonly used for all varieties, since these sequences are not exclusively present in the orange head cabbages. For example, the InDel primers (Bio130275/Bio130276) were designed based on a 6 bp deletion in the first exon for an orange head cabbage (A21530) [10]. However, this deletion is also detected in other white head cabbages (14-401 and 91-112) (Supplementary Material: File S2). The marker based on an 88 bp deletion in the promoter region of the *BrCRTISO* gene [11] cannot be utilized to determine the genotype in A21530, because in this cultivar, the gene does not have this deletion.

**Figure 4:**
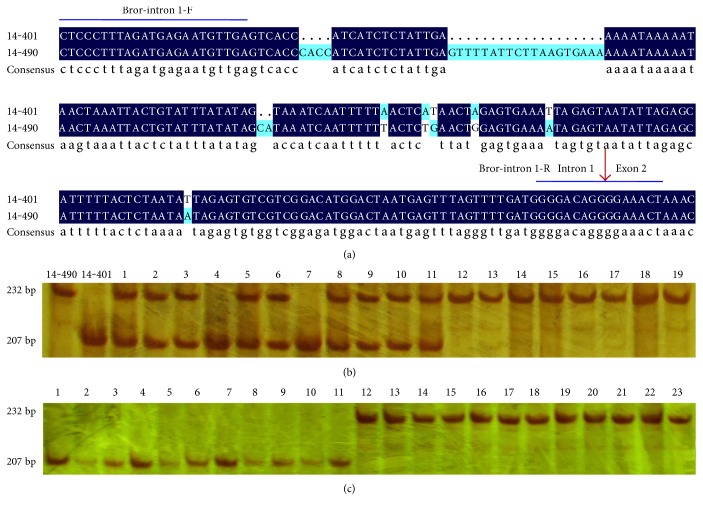
Validation of the gene-specific markers in different white and orange cultivars and F_2_ populations. (a) The amplified fragments by the Bror-intron 1-F/R primers in 14-401 and 14-490. The blue lines indicate where the Bror-intron 1-F/R primers bind. The red arrows represent the boundaries of the exon/intron. (b) Validation of the gene-specific marker Bror-intron 1 in the parents 14-401 and 14-490 and its F_2_ individuals. Among the F_2_ individuals, 1–11 are the lines with the white inner leaves, and 12–19 are the lines with the orange inner leaves. (c) Validation of the gene-specific marker Bror-intron 1 in the breeding lines. 1–11 are 663, 1466, 1469, 1492, 1505, 1510, 1720, Hanxiu, Jindianchunwang, Kaichun, and Ribenxiayang with the white or yellow inner leaves. 12–23 are 1480, 14-102, 14-245, 14-253, 14-257, 14-277, 14-426, 14-662, 14-669, Changyanjubao, Shenmengjuhongxin, and Shenshijuhongxin with the orange inner leaves.

In this study, based on the sequence difference of the *BrCRTISO* genes between14-401 and 14-490, we designed a pair of InDel primers (Bror-intron 1-F/R) (Figure 4(a); Supplementary Material: Table S1). Genotyping analysis of the *BrCRTISO* gene among the parents and F_2_ individuals revealed that this marker was cosegregated with the *Br-or* locus (Figure 4(b)). Additionally, this marker was applied to determine the genotypes of 23 Chinese cabbage varieties with different inner leaf colors. The results show that the cultivar genotypes identified by Bror-intron1 were consistent with the cultivar phenotypes (Figure 4(c)), suggesting that the sequence matched with Bror-intron1 is conserved in most orange head varieties, and this marker can be used to determine the Chinese cabbage genotypes at different developing stages.

## 4. Conclusion

Our results show that the *Br-or* gene varied significantly in different orange head cultivars. However, the C^952^ to T^952^ mutation in the *BrCRTISO* gene was largely conserved among the orange head cultivars, and this SNP was well cosegregated with the *Br-or* locus. Additionally, a new InDel molecular marker located in the first intron was developed. Both this InDel molecular marker and the SNP (C^952^ to T^952^) can be used in the marker-assisted breeding of the orange head Chinese cabbage with other beneficial traits. Furthermore, four genes related to carotenoid biosynthesis were downregulated, which could partially account for the accumulation of prolycopene and other pigments in the orange inner leaves. Further investigations, like *BrCRTISO* gene knockouts in the white head Chinese cabbage, are yet to be constructed to fully understand the mechanism underlying the pigment accumulation in the inner leaves.

## Conflicts of Interest

The authors declare that there is no conflict of interests regarding the publication of this paper.

## Supplementary Material

The information of supplementary materials are as follows: Table S1 Primers designed for real-time quantitative PCR, gene cloning, genome walking and DNA. File S1 Optimal alignment of the genome DNA sequences of Bra031539 from 14-490, 12-9 (Su et al, 2015) and Chiifu-401(from Brassica Database, BRAD). The underlined sequences represent the exons. File S2 Optimal alignment of the ORF sequences of Bra031539 from 14-490, 14-401, 12-9 and 91-112 (Su et al, 2015), A21530 and A21445 (Li et al, 2015) and Chiifu-401(from Brassica Database, BRAD). The red arrows represent the boundaries of the exons. Fig. S1 Validation of the SNP (C^952^ to T^952^) in different white and orange cultivars and F_2_ populations. A: Validation of the SNP (C^952^ to T^952^) in the parents 14-401 and 14-490, and its F_2_ individuals. Among the F_2_ individuals, 1-11 are the lines with the white inner leaves, and 12-19 are the lines with the orange inner leaves. B: Validation of the SNP (C^952^ to T^952^) in the breeding lines. 1-11 are 663, 1466, 1469, 1492, 1505, 1510, 1720, Hanxiu, Jindianchunwang, Kaichun and Ribenxiayang with the white or yellow inner leaves. 12-23 are 1480, 14-102, 14-245,14-253, 14-257, 14-277, 14-426, 14-662, 14-669, Changyanjubao, Shenmengjuhongxin and Shenshijuhongxin with the orange inner leaves. Table S2 Differentially expressed genes between the orange head and white head. File S3 Optimal alignment of the promoter sequences of Bra031539 from 14-401, 14-490, 12-9 and 91-112 (Su et al, 2014).

## Figures and Tables

**Table 1:** Summary of read numbers.

Read category	Map to annotation gene		Map to genome	
	Orange	White	Orange	White
Total reads	11,692,280	12,426,002	11,692,280	12,426,002
Total base pairs	572,921,720	608,874,098	572,921,720	608,874,098
Total mapped reads	7,816,773	8,342,444	9,609,140	10,261,712
Perfect match	5,729,753	6,140,776	7,003,617	7,518,873
≤2 bp mismatch	2,087,020	2,201,668	2,605,523	2,742,839
Unique match	7,308,446	7,787,139	8,607,533	9,166,275
Multiposition match	508,327	555,305	1,001,607	1,095,437
Total unmapped reads	3,875,507	4,083,558	2,083,140	2,164,290

**Table 2:** RNA-seq experiment validation by RT-qPCR. RPKM: reads per kb per million reads; FDR: false discovery rate; SE: standard error. The *p* value was calculated by the SPSS software.

Gene ID	RNA-seq			RT-qPCR		
	RPKM		FDR	2^-ΔCT^ (mean ± SE)		*p* value
	Orange	White		Orange	White	
Bra035683	9.18	3.68	2.30*E* − 05	0.0070 ± 0.00023	0.0073 ± 0.00115	0.812
Bra039047	0.09	4.26	2.03*E* − 11	0.0009 ± 0.00007	0.0426 ± 0.00036	0
Bra011759	1.54	10.23	1.13*E* − 16	0.0004 ± 0.00002	0.0189 ± 0.00185	0.01
Bra010598	0.99	6.14	2.31*E* − 09	0.0002 ± 0.00001	0.0112 ± 0.00068	0.004
Bra004735	0.81	4.43	0.000355	0.0005 ± 0.00004	0.0329 ± 0.00257	0.006
Bra025756	15.30	44.25	1.74*E* − 22	0.0020 ± 0.00021	0.0233 ± 0.00091	0
Bra031132	3.12	7.98	0.00027	0.0044 ± 0.00012	0.0067 ± 0.00015	0
Bra024643	6.58	16.11	2.31*E* − 07	0.0130 ± 0.00076	0.0232 ± 0.00178	0.006
Bra039555	10.39	23.40	1.82*E* − 10	0.0043 ± 0.00025	0.0199 ± 0.00313	0.037
Bra040203	8.33	16.68	8.20*E* − 05	0.0425 ± 0.00138	0.1340 ± 0.01970	0.01
Bra031539	2.71	3.34	0.732593	0.0155 ± 0.00059	0.0170 ± 0.00130	0.373

**Table 3:** KEGG pathway-enrichment analysis in DEGs (*Q* value ≤ 0.05).

Pathway ID	Pathway annotation	DEGs with pathway annotation	*Q* value
ko01110	Biosynthesis of secondary metabolites	Bra035683, Bra039047, Bra029560, Bra001454, Bra011759, Bra010598, Bra004735, Bra025756, Bra039945, Bra031132, Bra024643, Bra039555, Bra040203	0.000697023
ko00906	Carotenoid biosynthesis	Bra004735, Bra018969, Bra039945, Bra040203	0.001346228
ko00945	Stilbenoid, diarylheptanoid, and gingerol biosynthesis	Bra039047, Bra011759, Bra010598, Bra039555	0.011866805
ko00130	Ubiquinone and other terpenoid-quinone biosyntheses	Bra029560, Bra031132	0.032958714
ko00903	Limonene and pinene degradation	Bra039047, Bra011759, Bra010598	0.032958714
