# Supplementary material for: Transcriptome Analysis of Orange Head Chinese Cabbage (Brassica rapa L. ssp. pekinensis) and Molecular Marker Development
Source: Int J Genomics. 2017 Apr 2;2017:6835810. doi: 10.1155/2017/6835810 (PMC5392394; doi:10.1155/2017/6835810)
Supplement: Supplementary file 1 — The information of supplementary materials are as follows: Table S1 Primers designed for real-time quantitative PCR, gene cloning, genome walking and DNA. File S1 Optimal alignment of the genome DNA sequences of Bra031539 from 14-490, 12-9 (Su et al, 2015) and Chiifu-401(from Brassica Database, BRAD). The underlined sequences represent the exons. File S2 Optimal alignment of the ORF sequences of Bra031539 from 14-490, 14-401, 12-9 and 91-112 (Su et al, 2015), A21530 and A21445 (Li et al, 2015) and Chiifu-401(from Brassica Database, BRAD). The red arrows represent the boundaries of the exons. Fig. S1 Validation of the SNP (C952 to T952) in different white and orange cultivars and F2 populations. A: Validation of the SNP (C952 to T952) in the parents 14-401 and 14-490, and its F2 individuals. Among the F2 individuals, 1-11 are the lines with the white inner leaves, and 12-19 are the lines with the orange inner leaves. B: Validation of the SNP (C952 to T952) in the breeding lines. 1-11 are 663, 1466, 1469, 1492, 1505, 1510, 1720, Hanxiu, Jindianchunwang, Kaichun and Ribenxiayang with the white or yellow inner leaves. 12-23 are 1480, 14-102, 14-245,14-253, 14-257, 14-277, 14-426, 14-662, 14-669, Changyanjubao, Shenmengjuhongxin and Shenshijuhongxin with the orange inner leaves. Table S2 Differentially expressed genes between the orange head and white head. File S3 Optimal alignment of the promoter sequences of Bra031539 from 14-401, 14-490, 12-9 and 91-112 (Su et al, 2014). [file 6835810.f1.docx]

Table S1 Primers designed for real-time quantitative PCR, gene cloning, genome walking and DNA markers.

| Gene | Primer (5’-3’) |
| --- | --- |
| Primers for RT-qPCR  Bra035683  Bra039047  Bra011759  Bra010598  Bra004735  Bra025756  Bra031132  Bra024643  Bra039555  Bra040203  Bra031539  BrACTIN  Primers for gene cloning  Bra031539-P  Bra031539-G1  Bra031539-G2  Primers for genome walking  Bror-walking-1  Bror-walking-2  Bror-walking-3  Primers for DNA markers  Bror-intron1  Primers for validation of SNP^952^  OR-SNP^952^ | F: CAGTCCGAGGACGAACAAGG  R: GGTGGGAGATTCATGGAGGC  F: GCTCCAATGCTCCTCCCTCA  R: CAAGCCCTTCGTCCAATCCC  F: GCACCCTGGCGAATACTTACC  R: CCATCTTTATTTCCCCTACACTCAT  F: ACATTCCAAACCTGCCTTACCTC  R: GGACATCGTCCTTTTCTCCTTCT  F: TTCTGCGTGTTGTGGAACTGGA  R: TCGGTCGGATGAAAAGCGTC  F: ACCTCCCCAACCGTCGCC  R: GTCCAGTTTATCACTTTTCCAGCATC  F: CAGTTGAGGCTCTTGCAGATGACA  R: TGAAGATGCTGACGGGAGAAAAC  F: CTTGCCTGCGATTCTCGTTTG  R: GCTTCACAGCCAGTACATCCATACC  F: CGGACTTGGTCTGCTGAGGC  R: CGGTCTGCGGATTCTGGACT  F: AAATCTAGGGCTTTCTACGTCTTTGG  R: TAATGTCGGGGATGTTGAGGC  F: CTCTGTCTCCACAATCCCGTAAC  R: TGAGTCGCCGCAACTAATCCT  F: ATCCAGGCTGTTCTTTCCCTCTAC  R: TTCTCGCTCGGCGGTTGT  F: TTTTCTTGATGGAGTGAAAGGAGTC  R: GGTTCTGAGCTTGGTTCGTCGTCAC  F: ATGAATCTCTGTCTCCACAATCCCG  R: ACTAGTCCTCCTGCTAAAGACCTTG  F: GGTGTCTACCCCATGTTTCCC  R: CTATGCGAGTGTCCTTAACCAACCA  F: TATGGTAGGATTCTTGTTTTTGGG  F: GTTTCCCTTTCTGTTCAGTTTCTAA  F: AGTGTTCAAGGACAGGTTTTATGTG  F: CTCCCTTTAGATGAGAATGTTGA  R: AGTTTAGTTTCCCCTGTCCC  F: GATCCTCAGTTACTGTCTTTCAT  R: GAGTTATACAGAAACTTACAGCCT |
