# Supplementary material for: Transcriptome Analysis of Orange Head Chinese Cabbage (Brassica rapa L. ssp. pekinensis) and Molecular Marker Development
Source: Int J Genomics. 2017 Apr 2;2017:6835810. doi: 10.1155/2017/6835810 (PMC5392394; doi:10.1155/2017/6835810)
Supplement: Supplementary file 2 [file 6835810.f2.docx]

File S1 Optimal alignment of the genome DNA sequences of Bra031539 from 14-490, 12-9 (Su et al, 2015) and Chiifu-401(from Brassica Database, BRAD). The underlined sequences represent the exons.

12-9 ATGAATCTGTGTCTACACAATCCCGTAACGTGTGCTGATCGCAGCTCCTCCTTGTCATCG

14-490 ATGAATCTGTGTCTACACAATCCCGTAACGTGTGCTGATCGCAGCTCCTCCTTGTCATCG

Chiifu-401 ATGAATCTCTGTCTCCACAATCCCGTAACGTGTGCTGATCGCAGCTCCTCCTTGTTCTCC

******** ***** **************************************** **

12-9 GCCTTGAAGACTTCAAATTACAAACTGGGTACTTCAAAGTTTGGGTTTTTAAAGCATCGG

14-490 GCCTTGAAGACTTCAAATTACAAACTGGGTACTTCAAAGTTTGGGTTTTTAAAGCATCGG

Chiifu-401 GCCTTGAAGACTTCAAATAACAAGTTGGGTACTTCAAAGTTTGGGTTTTTAAAGAATCGG

****************** **** ***************************** *****

12-9 AAGAAGAATCATGTGGTTGCTGTGAGGTCTGTTTCTACGAGTACGGTC------ACTACC

14-490 AAGAAGAATCATGTGGTTGCTGTGAGGTCTGTTTCTACGAGTACGGTC------ACTACC

Chiifu-401 AAGAAGAATCATGTGGTTGCAGTGAGATCTGTTTCTTCCACTGCTGTAGAAGAAAGGACG

******************** ***** ********* * * * * ** * **

12-9 GTTAAAGAAGAGACGAAGAGAGAGAGTCAAGTGTACGACGCCATCGTCATCGGGTCTGGG

14-490 GTTAAAGAAGAGACGAAGAGAGAGAGTCAAGTGTACGACGCCATCGTCATCGGGTCTGGG

Chiifu-401 AAGAGAGAAAGTGGAGGAGGAGAGAGTAAAGTGTACGACGCAATCGTCATCGGGTCTGGG

* **** ******** ************* ******************

12-9 ATTGGAGGATTAGTTGCGGCGACTCAATTAGCTGTTAAAGAAGCTAAAGTTTTAGTTTTG

14-490 ATTGGAGGATTAGTTGCGGCGACTCAATTAGCTGTTAAAGAAGCTAAAGTTTTAGTTTTG

Chiifu-401 ATTGGAGGATTAGTTGCGGCGACTCAGCTAGCTGTTAAAGAAGCTAAAGTTTTAGTTTTG

************************** ********************************

12-9 GAGAAGTATCTGATCCCTGGTGGGAGCTCTGGTTACTACGAAAGAGATGGGTACACATTC

14-490 GAGAAGTATCTGATCCCTGGTGGGAGCTCTGGTTACTACGAAAGAGATGGGTACACATTC

Chiifu-401 GAGAAGTATCTGATCCCTGGTGGGAGCTCCGGTTATTACGAAAGAGATGGATACACATTC

***************************** ***** ************** *********

12-9 GATGTGGGTTCTTCTGTCATGTTTGGTTTCAGCGATAAGGTTCGTTTCGTTTGCTCTCGT

14-490 GATGTGGGTTCTTCTGTCATGTTTGGTTTCAGCGATAAGGTTCGTTTCGTTTGCTCTCGT

Chiifu-401 GATGTTGGCTCTTCTGTCATGTTTGGTTTCAGCGATAAGGTTCGTTTCATTTGCTCTCGT

***** ** *************************************** ***********

12-9 AAAGACTCTCCCTTTAGAT--------GAGAATGTTGAGTCACCCA-----CCATCATCT

14-490 AAAGACTCTCCCTTTAGAT--------GAGAATGTTGAGTCACCCA-----CCATCATCT

Chiifu-401 AAAGACTCTTCCTTTACATAATTATATGAGAATGTTGTGTCTTCCAGAAGACAATCATCT

********* ****** ** ********** *** *** * *******

12-9 CT----------ATTGAGTTATATTCTTAAGTGA---AAAAAATAAAAATAAGTAAATTA

14-490 CT----------ATTGAGTTATATTCTTAAGTGA---AAAAAATAAAAATAAGTAAATTA

Chiifu-401 CTCTATAACAGAATTGAGTTATAGTCTAATGAGACACGAAAAACAAAAATAAATAAATTA

** *********** *** * * ** ***** ******** *******

12-9 CTCTATTTATATAGCATAAATCAATTTTTTACTCTGTTATGGAGTGAAAATAGAGTAATA

14-490 CTCTATTTATATAGCATAAATCAATTTTTTACTCTGTTATGGAGTGAAAATAGAGTAATA

Chiifu-401 CTCTATTTATATAGCATAAACCAATTTTTAACTCATTTATAGAGTGAAAATAGAGCATTT

******************** ******** **** **** ************** * *

12-9 TTAGA-------GCATTTTTACTCTAAAAATAGAGTGTGGTCGGAGATGGACTAATGAGT

14-490 TTAGA-------GCATTTTTACTCTAAAAATAGAGTGTGGTCGGAGATGGACTAATGAGT

Chiifu-401 TTAATCTAAAATATATTCTAGATCGAAAAATAGAGTGGGGTTAGAGATGAACTAATGCTT

*** *** * ** ************ *** ****** ******* *

12-9 TTAGTTTT-GATGGGGACAGGGGAAACTAAACTTGATAACTCAGGCGTTGAAGGCAGTTG

14-490 TTAGTTTT-GATGGGGACAGGGGAAACTAAACTTGATAACTCAGGCGTTGAAGGCAGTTG

Chiifu-401 CTAGTTTTTGATGGGGACAGGGGAATCTAAACTTGATAACTCAAGCGTTGAAAGCAGTTG

******* **************** ***************** ******** *******

12-9 GTCGTGAGATGGAGGTTATACCTGATCCCACCACTGTCCATTTCCATCTTCCCAATGATC

14-490 GTCGTGAGATGGAGGTTATACCTGATCCCACCACTGTCCATTTCCATCTTCCCAATGATC

Chiifu-401 GTCGTGAGATGGAGGTTATACCTGATCCCACCACCGTTCATTTCCATCTTCCCAATGATC

********************************** ** **********************

12-9 TCTCTGTTCAGGTTCATAGAGAGTATGATGAGTTCGTTAATGAGCTTATTAGCAAGTTTC

14-490 TCTCTGTTCAGGTTCATAGAGAGTATGATGAGTTCGTTAATGAGCTTATTAGCAAGTTTC

Chiifu-401 TCTCTGTTCAGGTTCATAGAGAGTATGATGAGTTCGTTAATGAGCTTATTAGCAAGTTTC

************************************************************

12-9 CGCACGAGAAGGAAGGGATTCTTGGATTCTATGGCGTCTGCTGGAAGGTTCTGATGTTGC

14-490 CGCACGAGAAGGAAGGGATTCTTGGATTCTATGGCGTCTGCTGGAAGGTTCTGATGTTGC

Chiifu-401 CGCACGAGAAGGAAGGGATTCTTGGATTCTATGGCATCTGCTGGAAGGTTCTGATGTGAC

*********************************** ********************* *

12-9 TTTTTTACTTAATTGAATATTTGTAAGAGTGTGTA-TTTAAAGATTACAGATTGGAACAG

14-490 TTTTTTACTTAATTGAATATTTGTAAGAGTGTGTA-TTTAAAGATTACAGATTGGAACAG

Chiifu-401 TTTT--ACTTAATTGAATAGTTGTAAGAGTGTGTAATTTAAATATTACAAATTGGAACAG

**** ************* *************** ****** ****** **********

12-9 ATCTTCAACTCATTGAACTCTTTGGAACTGAAGTCGCTTGAAGAGCCTATCTACCTTTTT

14-490 ATCTTCAACTCATTGAACTCTTTGGAACTGAAGTCGCTTGAAGAGCCTATCTACCTTTTT

Chiifu-401 ATCTTCAACTCATTGAACTCTTTGGAACTGAAGTCGCTTGAAGAGCCTATCTACCTTTTT

************************************************************

12-9 GGACAGTTCTTTCAGAAGCCCCTTGAATGCTTGACACTCGGTACTCTTTTTGATGTTTGT

14-490 GGACAGTTCTTTCAGAAGCCCCTTGAATGCTTGACACTCGGTACTCTTTTTGATGTTTGT

Chiifu-401 GGACAGTTCTTTCAGAAGCCGCTTGAATGCTTGACACTCGGTACTCTTTTTGATGTTTGT

******************** ***************************************

12-9 TTGTGTATTTATTAGTTTCTGTGAGTGTATACATTGAATCTTTTTATATTATTCTCTGTG

14-490 TTGTGTATTTATTAGTTTCTGTGAGTGTATACATTGAATCTTTTTATATTATTCTCTGTG

Chiifu-401 TTGTGTATTTATTAGTTTCTGTGAGTGTATACATTGAATCTTTTTATATTATTCTCTGTG

************************************************************

12-9 ATTTATGTACAGCTTATTACTTGCCTCAAAATGCTGGGGATATAGCTCGGAAGTACATAA

14-490 ATTTATGTACAGCTTATTACTTGCCTCAAAATGCTGGGGATATAGCTCGGAAGTACATAA

Chiifu-401 ATTTATGTACAGCTTATTACTTGCCTCAAAATGCTGGGGATATAGCTCGGAAGTACATAA

************************************************************

12-9 AGGATCCTCAGTTACTGTCTTTCATTGACGCAGAGGTTAGCAAAAAATGTCATTTGGTCC

14-490 AGGATCCTCAGTTACTGTCTTTCATTGACGCAGAGGTTAGCAAAAAATGTCATTTGGTCC

Chiifu-401 AGGATCCTCAGTTACTGTCTTTCATTGACGCAGAGGTTAGCAAAAAATGTCATTTGGTCC

************************************************************

12-9 AAACATGCACAGTTTATTTTACACCATTTGTATTTTTTGCCTGCAGTGTTTCATTGTGAG

14-490 AAACATGCACAGTTTATTTTACACCATTTGTATTTTTTGCCTGCAGTGTTTCATTGTGAG

Chiifu-401 AAACATGCACAGTTTATTTTACACCATTTGTATTTATT-CCTGCAGTGTTTCATTGTGAG

*********************************** ** *********************

12-9 CACAGTGAATGCTTTGCAGACGCCGATGATCAATGCAAGTATGGTAGGATTCTTGTTTTT

14-490 CACAGTGAATGCTTTGCAGACGCCGATGATCAATGCAAGTATGGTAGGATTCTTGTTTTT

Chiifu-401 TACAGTCAATGCTTTGCAGACGCCAATGATCAATGCAAGTATGGTAGGATTCTTGTTTTT

***** ***************** ***********************************

12-9 GGGCATTTTGGTGTCTACTCCATGTTTCCCTTTCTGTTCAGTTTCTAAGTGTTCAAGGAC

14-490 GGGCATTTTGGTGTCTACTCCATGTTTCCCTTTCTGTTCAGTTTCTAAGTGTTCAAGGAC

Chiifu-401 GGGCATTTTGGTGTCTACCCCATGTTTCCCTTTCTGTTCAGTTTCTAAGTGTTCAAGGAC

****************** *****************************************

12-9 AGGTTTTATGTGACAGGCACTATGGAGGGATTAACTACCCTGTTGGTGGTGTTGGTGGGA

14-490 AGGTTTTATGTGACAGGCACTATGGAGGGATTAACTACCCTGTTGGTGGTGTTGGTGGGA

Chiifu-401 AGGTTTTATGTGACAGGCACTATGGAGGGATTAACTACCCTGTTGGTGGTGTTGGTGGGA

************************************************************

12-9 TTGCAAGGTCTTTAGCAGGAGGACTAGTTGATCAAGGAAGTGAAATATTCTACAAAGCTA

14-490 TTGCAAGGTCTTTAGCAGGAGGACTAGTTGATCAAGGAAGTGAAATATTCTACAAAGCTA

Chiifu-401 TTGCAAGGTCTTTAGCAGGAGGACTAGTTGATCAAGGAAGTGAAATACTCTACAAAGCTA

*********************************************** ************

12-9 ATGTGAAAAGCATAATTCTTGATGATGGAAAGGCTGTAAGTTTCTGTATAACTCTTGCTA

14-490 ATGTGAAAAGCATAATTCTTGATGATGGAAAGGCTGTAAGTTTCTGTATAACTCTTGCTA

Chiifu-401 ATGTGAAAAGCATAATTCTTGATGATGGAAAGGCTGTAAGTTTCTGTATAACTCTTGCTA

************************************************************

12-9 CATTGATTCCAAGTTGTTGTTCTAAAAATCGATCTAGACCTCCGACTAGTCAGCAAATCA

14-490 CATTGATTCCAAGTTGTTGTTCTAAAAATCGATCTAGACCTCCGACTAGTCAGCAAATCA

Chiifu-401 CATATATTCCAAGTTGTTGTTCTAAAACTCGATC------------TAGTCAGCAAATCA

*** ********************** ****** **************

12-9 GTCCTAAAGAAAACAATTTT--------TCGGTTTAGATGCTCAAAATATCGGTCTAGGC

14-490 GTCCTAAAGAAAACAATTTT--------TCGGTTTAGATGCTCAAAATATCGGTCTAGGC

Chiifu-401 GACCTAAAGGAAACAATTTTAAAAAAAATCGGTTTAGATGCTCAAAATATCGTTCTAGGC

* ******* ********** ************************ *******

12-9 GCCCGCATAATCAATAATCCCATATAAAGCTTCCAACTACCCCTACCG--TTCTTGAACA

14-490 GCCCGCATAATCAATAATCCCATATAAAGCTTCCAACTACCCCTACCG--TTCTTGAACA

Chiifu-401 GCCCGCCTAATCAATAATCCCATATAAAGCTTCCAACTACCCCTACCGAGTTCTTGAACA

****** ***************************************** **********

12-9 TTGATTCTTGCTTAACCTCTTGAAATTCTTATTTGGTTGTGTTTTCT----ATCTTCAGG

14-490 TTGATTCTTGCTTAACCTCTTGAAATTCTTATTTGGTTGTGTTTTCT----ATCTTCAGG

Chiifu-401 TTGATTCTTGCTTAACCTCTTGAAATTCTTATTTGGTTGTGTTTTTTTTTTATCTTCAGG

********************************************* * *********

12-9 TGGGTGTAAGGCTAGCAGATGGAAGAGAATTCTTCGCTAAAACGATAATTTCTAATGCTA

14-490 TGGGTGTAAGGCTAGCAGATGGAAGAGAATTCTTCGCTAAAACGATAATTTCTAATGCTA

Chiifu-401 TGGGTGTAAGGCTAGCAGATGGAAGAGAGTTCTTCGCTAAAACAATAATTTCTAATGCTA

**************************** ************** ****************

12-9 CAAGATGGGATACGTTTGGTAAGAGAAAA--CAATGACTTGCCTAAGTGTTGAATAGGTG

14-490 CAAGATGGGATACGTTTGGTAAGAGAAAA--CAATGACTTGCCTAAGTGTTGAATAGGTG

Chiifu-401 CAAGATGGGATACGTTTGGTAAGAGAAAAGACAATGACTTACCAAACTGTTGCATAAGTG

***************************** ********* ** ** ***** *** ***

12-9 TCTTGTTTGGTAAGAATACTT-AGCATAGTATTACTAATGATGTAGGGAAGCTGTTGAAA

14-490 TCTTGTTTGGTAAGAATACTT-AGCATAGTATTACTAATGATGTAGGGAAGCTGTTGAAA

Chiifu-401 TTTTGTTCTGTAAGAATACTTTAACATAGTATTA-TGGTGCTGTAGGGAAGCTGTTGAAA

* ***** ************ * ********** * ** *******************

12-9 GGAGAAAAGCTTCCAAAAGAAGAAGAAAACTTCCAGAAAGTCTACGTGAAGGCTCCATCG

14-490 GGAGAAAAGCTTCCAAAAGAAGAAGAAAACTTCCAGAAAGTCTACGTGAAGGCTCCATCG

Chiifu-401 GGAGAAAAGCTTCCAAAAGAAGAAGAAAACTTCCAGAAAGTCTATGTGAAGGCTCCATCG

******************************************** ***************

12-9 TTTCTTTCAATCCACATGGGTGTTAAAGCAGAGGTTCTCCCTCCAGATACAGATTGCCAT

14-490 TTTCTTTCAATCCACATGGGTGTTAAAGCAGAGGTTCTCCCTCCAGATACAGATTGCCAT

Chiifu-401 TTTCTCTCAATCCACATGGGTGTTAAAGCAGAGGTTCTCCCTCCAGATACAGATTGCCAT

***** ******************************************************

12-9 CATTTCGTACTTGAGGTTTGTTAGTTTCTTACTAAGATCTATTATTCCCTTTGACAGATT

14-490 CATTTCGTACTTGAGGTTTGTTAGTTTCTTACTAAGATCTATTATTCCCTTTGACAGATT

Chiifu-401 CATTTCGTACTTGAGGTTTGTTAGTTTCTTACTAAGATCTATTATTCCCTTTGACAGATT

************************************************************

12-9 CATAGTTATTTGAATAGTGGTTATGTTCTTTTGTTGCAACCTGTTAGGATGATTGGAAGA

14-490 CATAGTTATTTGAATAGTGGTTATGTTCTTTTGTTGCAACCTGTTAGGATGATTGGAAGA

Chiifu-401 CATAGTTATTTGAATAGTGGTTATGTTCTTTTGTTGCAACCTGTTAGGATGATTGGAAGA

************************************************************

12-9 ATCTGGAGGAGCCTTATGGCAGTATCTTCCTCAGCATCCCAACCATTCTTGATCCATCCT

14-490 ATCTGGAGGAGCCTTATGGCAGTATCTTCCTCAGCATCCCAACCATTCTTGATCCATCCT

Chiifu-401 ATCTGGAGGAGCCTTATGGCAGTATCTTCCTCAGCATCCCAACCATTCTTGATCCATCCT

************************************************************

12-9 TGGCTCCAGATGGTCGACATATACTCCACATATTTACAACTTCTTCCATTGAAGATTGGG

14-490 TGGCTCCAGATGGTCGACATATACTCCACATATTTACAACTTCTTCCATTGAAGATTGGG

Chiifu-401 TGGCTCCAGATGGTCGACATATACTCCACATATTTACAACTTCTTCCATTGAAGATTGGG

************************************************************

12-9 AGGTAAGAGGCTGATCTTAAAAGAGTGAGACCAAGCATCAATTATATATGCCATCTTATT

14-490 AGGTAAGAGGCTGATCTTAAAAGAGTGAGACCAAGCATCAATTATATATGCCATCTTATT

Chiifu-401 AGGTAAGAGGCTGATCTTAAAAGAGTGAGACCAAGCATCAATTATATATGCCATCTTATT

************************************************************

12-9 TGACCGTTTCATCTAAATTTTGGTACTATAGGGACTCACTCCAAAAGAGTATGAGGCTAA

14-490 TGACCGTTTCATCTAAATTTTGGTACTATAGGGACTCACTCCAAAAGAGTATGAGGCTAA

Chiifu-401 TGACCGTTTCATCTAAATTTTGGTACTATAGGGACTCACTCCAAAAGAGTATGAGGCTAA

************************************************************

12-9 AAAAGAAGAGGTGGCAGCTGGAATCATACAGAGGCTAGAGAAAAAACTGTTTCCTGGGCT

14-490 AAAAGAAGAGGTGGCAGCTGGAATCATACAGAGGCTAGAGAAAAAACTGTTTCCTGGGCT

Chiifu-401 AAAAGAAGAGGTGGCAGCTGGAATCATACAGAGGCTAGAGAAAAAACTGTTTCCTGGGCT

************************************************************

12-9 CAGTTCATCTATTACTTTTAAGGAGGTTAGACAATCTATGTTCCTGCCTAGATTTGCCAA

14-490 CAGTTCATCTATTACTTTTAAGGAGGTTAGACAATCTATGTTCCTGCCTAGATTTGCCAA

Chiifu-401 CAGTTCATCTATTACTTTTAAGGAGGTTAGACAATCTATGTTCCTGCCTAGATTTGCCAA

************************************************************

12-9 TTCACATCTGTATATCTAAGGTGTTTTTATGAGGCAGGTGGGCACACCAAGAACACACAG

14-490 TTCACATCTGTATATCTAAGGTGTTTTTATGAGGCAGGTGGGCACACCAAGAACACACAG

Chiifu-401 TTCACATCTGTATATCTAAGGTGTTTTTATGAGGCAGGTGGGCACACCAAGAACACACAG

************************************************************

12-9 GCGATATCTTGCTAGGGATAAGGGAACGTATGGACCAATGCCAAGAGGAACACCAAAAGG

14-490 GCGATATCTTGCTAGGGATAAGGGAACGTATGGACCAATGCCAAGAGGAACACCAAAAGG

Chiifu-401 GCGATATCTTGCTAGGGATAAGGGAACGTATGGACCAATGCCAAGAGGAACACCAAAAGG

************************************************************

12-9 TTTACTAGGCATGCCGTTTAACACAACTGTAAGTCAAAAGAAAAGATTAGATGGTTCCCT

14-490 TTTACTAGGCATGCCGTTTAACACAACTGTAAGTCAAAAGAAAAGATTAGATGGTTCCCT

Chiifu-401 TTTACTAGGCATGCCGTTTAACACAACTGTAAGTCAAAAGAAACGATTAGATGGTTCCCT

******************************************* ****************

12-9 TCTGGGGCTTAACTAAATGGTTTTGGCTGATTGTAATAGGCTATAGATGGTTTGTACTGC

14-490 TCTGGGGCTTAACTAAATGGTTTTGGCTGATTGTAATAGGCTATAGATGGTTTGTACTGC

Chiifu-401 TCTTGGGCTTAACTAAATGGTTTTGGCTGATTGTAATAGGCTATAGATGGTTTGTACTGC

*** ********************************************************

12-9 GTTGGGGATAGTTGTTTTCCTGGTCAGGGAGTTATAGCTGTGGCTTTCTCAGGAGTGATG

14-490 GTTGGGGATAGTTGTTTTCCTGGTCAGGGAGTTATAGCTGTGGCTTTCTCAGGAGTGATG

Chiifu-401 GTTGGGGATAGTTGTTTTCCTGGTCAGGGAGTTATAGCTGTGGCTTTCTCAGGAGTGATG

************************************************************

12-9 TGTGCTCATCGTGTAGCTGCTGACATTGGTGAGAAAATTTGTTATAGACACTTGTTGATT

14-490 TGTGCTCATCGTGTAGCTGCTGACATTGGTGAGAAAATTTGTTATAGACACTTGTTGATT

Chiifu-401 TGTGCTCATCGTGTAGCTGCTGACATTGGTGAGAAAATTTGTAATAGAGACTTGTTGATT

****************************************** ***** ***********

12-9 GCTATGAGAGTAGGGATGTTA-ACGTGGGTAAAATAACCCAGCCCAGCCCAACCCACAAA

14-490 GCTATGAGAGTAGGGATGTTA-ACGTGGGTAAAATAACCCAGCCCAGCCCAACCCACAAA

Chiifu-401 GCTATGAGAGTAAGAAGCTAACATTTTTTTTTCATATTTTGATTTGTAGGGCTTGAGAGA

************ * * * * * * * *** * * *

12-9 TAACCCAACCCAGTTTATACCCAACCTGCAAAAACCCAGAAACATCAGGGTTGAAATCTA

14-490 TAACCCAACCCAGTTTATACCCAACCTGCAAAAACCCAGAAACATCAGGGTTGAAATCTA

Chiifu-401 AAATCAAAGGTACTTGATGCTGGTCTT--------CTTGGTTTACTTGGTTGGTTAAGGA

** * ** * ** ** * * * * * * ** * * * *

12-9 AACCCAGAAAATAAACCCAATATGGTATAGGTTTACCCGTGGGTACCCAAAGTATTATCT

14-490 AACCCAGAAAATAAACCCAATATGGTATAGGTTTACCCGTGGGTACCCAAAGTATTATCT

Chiifu-401 CACTCGCATAG-------------------------------------------------

** * * *

12-9 TATTTATTCTGAAGATCATGTAAAACTCATTTATGGTTTTAACGAGAAAACTTGTAAAGT

14-490 TATTTATTCTGAAGATCATGTAAAACTCATTTATGGTTTTAACGAGAAAACTTGTAAAGT

Chiifu-401 ------------------------------------------------------------

12-9 TGTTTTTGTGGTTTTAGCGGAAATTTTTCTTTTTGCGGTTTTTGGTCGGTAATTTTATTT

14-490 TGTTTTTGTGGTTTTAGCGGAAATTTTTCTTTTTGCGGTTTTTGGTCGGTAATTTTATTT

Chiifu-401 ------------------------------------------------------------

12-9 TGTGGCTTGGTTGGAAAACTCATTTTTGCGGTTTGCGGGAAAAATAATCTTTCTGGTTTT

14-490 TGTGGCTTGGTTGGAAAACTCATTTTTGCGGTTTGCGGGAAAAATAATCTTTCTGGTTTT

Chiifu-401 ------------------------------------------------------------

12-9 GACGAAAAAATTCGGTTTTACGGTTTTTGCGAGAAAATTCGGTTTAGCAGTTTTGGCAGG

14-490 GACGAAAAAATTCGGTTTTACGGTTTTTGCGAGAAAATTCGGTTTAGCAGTTTTGGCAGG

Chiifu-401 ------------------------------------------------------------

12-9 AAACCTCGCTTTTGCGGTTTTGGCGGAAAAACTCGTTTTTGATTTTGACGGAAAAACTTG

14-490 AAACCTCGCTTTTGCGGTTTTGGCGGAAAAACTCGTTTTTGATTTTGACGGAAAAACTTG

Chiifu-401 ------------------------------------------------------------

12-9 TTTTTACGGTTTTGGGGAAACTCGGTTTTCGGCTTTGACGGGAAAACTCGATTTTTCGAT

14-490 TTTTTACGGTTTTGGGGAAACTCGGTTTTCGGCTTTGACGGGAAAACTCGATTTTTCGAT

Chiifu-401 ------------------------------------------------------------

12-9 TTTGGCGGGAAAACTCGATTTTGCGGTTTTGGCGGGAAAACTCGGTTTTTCTGTTTTGGC

14-490 TTTGGCGGGAAAACTCGATTTTGCGGTTTTGGCGGGAAAACTCGGTTTTTCTGTTTTGGC

Chiifu-401 ------------------------------------------------------------

12-9 GGAAAAACCATGTTTTTCGCTTTCGGCAGTAAAAATCGTTTTGGCGGGAAAATTGAGTTT

14-490 GGAAAAACCATGTTTTTCGCTTTCGGCAGTAAAAATCGTTTTGGCGGGAAAATTGAGTTT

Chiifu-401 ------------------------------------------------------------

12-9 TACGGCATTGGCGGGAAAACACCTTTTGCGGTTTTGGCGGAAAAACTCGATTTTGGGGCT

14-490 TACGGCATTGGCGGGAAAACACCTTTTGCGGTTTTGGCGGAAAAACTCGATTTTGGGGCT

Chiifu-401 ------------------------------------------------------------

12-9 TTCAGTCGGAAAACTCGATTTTACGGTTTTAGCGGGAAAACTCAGTTTTGCAGTTTTGGT

14-490 TTCAGTCGGAAAACTCGATTTTACGGTTTTAGCGGGAAAACTCAGTTTTGCAGTTTTGGT

Chiifu-401 ------------------------------------------------------------

12-9 GAGAAAACTCAGTTTTGCGGTTTTGGCGGGAAACTTAGTTTTATGGTTTTGGCGGAAAAA

14-490 GAGAAAACTCAGTTTTGCGGTTTTGGCGGGAAACTTAGTTTTATGGTTTTGGCGGAAAAA

Chiifu-401 ------------------------------------------------------------

12-9 CAAGTTTTGTGGTTTCGGTAGAAAACCTCGATTTTTCGGTTTCGGCGGGAAAACTCGTTT

14-490 CAAGTTTTGTGGTTTCGGTAGAAAACCTCGATTTTTCGGTTTCGGCGGGAAAACTCGTTT

Chiifu-401 ------------------------------------------------------------

12-9 TTGGTTTCTGTGA

14-490 TTGGTTTCTGTGA

Chiifu-401 -------------
