# Supplementary material for: Transcriptome Analysis of Orange Head Chinese Cabbage (Brassica rapa L. ssp. pekinensis) and Molecular Marker Development
Source: Int J Genomics. 2017 Apr 2;2017:6835810. doi: 10.1155/2017/6835810 (PMC5392394; doi:10.1155/2017/6835810)
Supplement: Supplementary file 3 [file 6835810.f3.docx]

File S2 Optimal alignment of the ORF sequences of Bra031539 from 14-490, 14-401, 12-9 and 91-112 (Su et al, 2015), A21530 and A21445 (Li et al, 2015) and Chiifu-401(from Brassica Database, BRAD). The red arrows represent the boundaries of the exons.

14-490 ATGAATCTGTGTCTACACAATCCCGTAACGTGTGCTGATCGCAGCTCCTCCTTGTCATCG

12-9 ATGAATCTGTGTCTACACAATCCCGTAACGTGTGCTGATCGCAGCTCCTCCTTGTCATCG

A21530 ATGAATCTCTGTCTCCACAATCCCGTAACGTGTGCTGATCGCAGCTCCTCCTTGTCATCG

14-401 ATGAATCTCTGTCTCCACAATCCCGTAACGTGTGCTGATCGCAGCTCCTCCTTGTCATCG

91-112 ATGAATCTCTGTCTCCACAATCCCGTAACGTGTGCTGATCGCAGCTCCTCTTTGTTCTCC

A21445 ATGAATCTCTGTCTCCACAATCCCGTAACGTGTGCTGATCGCAGCTCCTCCTTGTTCTCC

Chiifu-401 ATGAATCTCTGTCTCCACAATCCCGTAACGTGTGCTGATCGCAGCTCCTCCTTGTTCTCC

******** ***** *********************************** **** **

14-490 GCCTTGAAGACTTCAAATTACAAACTGGGTACTTCAAAGTTTGGGTTTTTAAAGCATCGG

12-9 GCCTTGAAGACTTCAAATTACAAACTGGGTACTTCAAAGTTTGGGTTTTTAAAGCATCGG

A21530 GCCTTGAAGACTTCAAATTACAAACTGGGTACTTCAAAGTTTGGGTTTTTAAAGCATCGG

14-401 GCCTTGAAGACTTCAAATTACAAACTGGGTACTTCAAAGTTTGGGTTTTTAAAGCATCGG

91-112 GCCTTGAAGACTTCAAATTACAAACTGGGTACTTCAAAGTTTGGGTTTTTAAAGCATCGG

A21445 GCCTTGAAGACTTCAAATAACAAGTTGGGTACTTCAAAGTTTGGGTTTTTAAAGAATCGG

Chiifu-401 GCCTTGAAGACTTCAAATAACAAGTTGGGTACTTCAAAGTTTGGGTTTTTAAAGAATCGG

****************** **** ***************************** *****

14-490 AAGAAGAATCATGTGGTTGCTGTGAGGTCTGTTTCTACGAGTACGGTC------ACTACC

12-9 AAGAAGAATCATGTGGTTGCTGTGAGGTCTGTTTCTACGAGTACGGTC------ACTACC

A21530 AAGAAGAATCATGTGGTTGCTGTGAGGTCTGTTTCTACGAGTACGGTC------ACTACC

14-401 AAGAAGAATCATGTGGTTGCTGTGAGGTCTGTTTCTACGAGTACGGTC------ACTACC

91-112 AAGAAGAATCATGTGGTTGCTGTGAGGTCTGTTTCTACGAGTACGGTC------ACTACC

A21445 AAGAAGAATCATGTGGTTGCAGTGAGATCTGTTTCTTCCACTGCTGTAGAAGAAAGGACG

Chiifu-401 AAGAAGAATCATGTGGTTGCAGTGAGATCTGTTTCTTCCACTGCTGTAGAAGAAAGGACG

******************** ***** ********* * * * * ** * **

14-490 GTTAAAGAAGAGACGAAGAGAGAGAGTCAAGTGTACGACGCCATCGTCATCGGGTCTGGG

12-9 GTTAAAGAAGAGACGAAGAGAGAGAGTCAAGTGTACGACGCCATCGTCATCGGGTCTGGG

A21530 GTTAAAGAAGAGACGAAGAGAGAGAGTCAAGTGTACGACGCCATCGTCATCGGGTCTGGG

14-401 GTTAAAGAAGAGACGAAGAGAGAGAGTCAAGTGTACGACGCCATCGTCATCGGGTCTGGG

91-112 GTTAAAGAAGAGACGAAGAGAGAGAGTCAAGTGTACGACGCCATCGTCATCGGGTCTGGG

A21445 AAGAGAGAAAGTGGAGGAGGAGAGAGTAAAGTGTACGACGCAATCGTCATCGGGTCTGGG

Chiifu-401 AAGAGAGAAAGTGGAGGAGGAGAGAGTAAAGTGTACGACGCAATCGTCATCGGGTCTGGG

* **** ******** ************* ******************

14-490 ATTGGAGGATTAGTTGCGGCGACTCAATTAGCTGTTAAAGAAGCTAAAGTTTTAGTTTTG

12-9 ATTGGAGGATTAGTTGCGGCGACTCAATTAGCTGTTAAAGAAGCTAAAGTTTTAGTTTTG

A21530 ATTGGAGGATTAGTTGCGGCGACTCAATTAGCTGTTAAAGAAGCTAAAGTTTTAGTTTTG

14-401 ATTGGAGGATTAGTTGCGGCGACTCAGCTAGCAGTTAAAGAAGCTAAAGTTCTAGTTTTG

91-112 ATTGGAGGATTAGTTGCGGCGACTCAGCTAGCTGTTAAAGAAGCTAAAGTTTTAGTTTTG

A21445 ATTGGAGGATTAGTTGCGGCGACTCAGCTAGCTGTTAAAGAAGCTAAAGTTTTAGTTTTG

Chiifu-401 ATTGGAGGATTAGTTGCGGCGACTCAGCTAGCTGTTAAAGAAGCTAAAGTTTTAGTTTTG

************************** **** ****************** ********

14-490 GAGAAGTATCTGATCCCTGGTGGGAGCTCTGGTTACTACGAAAGAGATGGGTACACATTC

12-9 GAGAAGTATCTGATCCCTGGTGGGAGCTCTGGTTACTACGAAAGAGATGGGTACACATTC

A21530 GAGAAGTATCTGATCCCTGGTGGGAGCTCTGGTTACTACGAAAGAGATGGGTACACATTC

14-401 GAGAAGTATCTGATCCCTGGTGGGAGCTCCGGTTATTACGAAAGAGATGGATACACATTC

91-112 GAGAAGTATCTGATCCCTGGTGGGAGCTCCGGTTATTACGAAAGAGATGGATACACATTC

A21445 GAGAAGTATCTGATCCCTGGTGGGAGCTCCGGTTATTACGAAAGAGATGGATACACATTC

Chiifu-401 GAGAAGTATCTGATCCCTGGTGGGAGCTCCGGTTATTACGAAAGAGATGGATACACATTC

***************************** ***** ************** *********

14-490 GATGTGGGTTCTTCTGTCATGTTTGGTTTCAGCGATAAGGGGAAACTAAACTTGATAACT

12-9 GATGTGGGTTCTTCTGTCATGTTTGGTTTCAGCGATAAGGGGAAACTAAACTTGATAACT

A21530 GATGTGGGTTCTTCTGTCATGTTTGGTTTCAGCGATAAGGGGAATCTAAACTTGATAACT

14-401 GATGTGGGTTCCTCTGTCATGTTTGGTTTCAGCGATAAGGGAAAACTAAACTTGATAACT

91-112 GATGTTGGCTCTTCTGTCATGTTTGGTTTCAGCGATAAGGGGAAACTAAACTTGATAACT

A21445 GATGTTGGCTCTTCTGTCATGTTTGGTTTCAGCGATAAGGGGAATCTAAACTTGATAACT

Chiifu-401 GATGTTGGCTCTTCTGTCATGTTTGGTTTCAGCGATAAGGGGAATCTAAACTTGATAACT

***** ** ** ***************************** ** ***************

14-490 CAGGCGTTGAAGGCAGTTGGTCGTGAGATGGAGGTTATACCTGATCCCACCACTGTCCAT

12-9 CAGGCGTTGAAGGCAGTTGGTCGTGAGATGGAGGTTATACCTGATCCCACCACTGTCCAT

A21530 CAAGCGTTGAAAGCAGTTGGTCGTGAGATGGAGGTTATACCTGATCCCACCACCGTTCAT

14-401 CAGGCGTTGAAGGCAGTTGGTCGTGAGATGGAGGTTATACCTGATCCCACCACCGTTCAT

91-112 CAGGCGTTGAAGGCAGTTGGTCGTGAGATGGAGGTTATACCTGATCCCACCACTGTTCAT

A21445 CAAGCGTTGAAAGCAGTTGGTCGTGAGATGGAGGTTATACCTGATCCCACCACCGTTCAT

Chiifu-401 CAAGCGTTGAAAGCAGTTGGTCGTGAGATGGAGGTTATACCTGATCCCACCACCGTTCAT

** ******** ***************************************** ** ***

14-490 TTCCATCTTCCCAATGATCTCTCTGTTCAGGTTCATAGAGAGTATGATGAGTTCGTTAAT

12-9 TTCCATCTTCCCAATGATCTCTCTGTTCAGGTTCATAGAGAGTATGATGAGTTCGTTAAT

A21530 TTCCATCTTCCCAATGATCTCTCTGTTCAGGTTCATAGAGAGTATGATGAGTTCGTTAAT

14-401 TTCCATCTTCCCAATGATCTCTCTGTTCAGGTTCATAGAGAGTATGATGAGTTCGTTAAT

91-112 TTCCATCTTCCCAATGATCTCTCTGTTCAGGTTCATAGAGAGTATGATGAGTTCGTTAAT

A21445 TTCCATCTTCCCAATGATCTCTCTGTTCAGGTTCATAGAGAGTATGATGAGTTCGTTAAT

Chiifu-401 TTCCATCTTCCCAATGATCTCTCTGTTCAGGTTCATAGAGAGTATGATGAGTTCGTTAAT

************************************************************

14-490 GAGCTTATTAGCAAGTTTCCGCACGAGAAGGAAGGGATTCTTGGATTCTATGGCGTCTGC

12-9 GAGCTTATTAGCAAGTTTCCGCACGAGAAGGAAGGGATTCTTGGATTCTATGGCGTCTGC

A21530 GAGCTTATTAGCAAGTTTCCGCACGAGAAGGAAGGGATTCTTGGATTCTATGGCATCTGC

14-401 GAGCTTATTAGCAAGTTTCCGCACGAGAAGGAAGGGATTCTTGGATTCTATGGCATCTGC

91-112 GAGCTTATTAGCAAGTTTCCGCACGAGAAGGAAGGGATTCTTGGATTCTATGGCGTCTGC

A21445 GAGCTTATTAGCAAGTTTCCGCACGAGAAGGAAGGGATTCTTGGATTCTATGGCATCTGC

Chiifu-401 GAGCTTATTAGCAAGTTTCCGCACGAGAAGGAAGGGATTCTTGGATTCTATGGCATCTGC

****************************************************** *****

14-490 TGGAAGATCTTCAACTCATTGAACTCTTTGGAACTGAAGTCGCTTGAAGAGCCTATCTAC

12-9 TGGAAGATCTTCAACTCATTGAACTCTTTGGAACTGAAGTCGCTTGAAGAGCCTATCTAC

A21530 TGGAAGATCTTCAACTCATTGAACTCTTTGGAACTGAAGTCGCTTGAAGAGCCTATCTAC

14-401 TGGAAGATCTTCAACTCATTGAACTCTTTGGAACTGAAGTCGCTTGAAGAGCCTATCTAC

91-112 TGGAAGATCTTCAACTCATTGAACTCTTTGGAACTCAAGTCTCTTGAAGAACCTATCTAC

A21445 TGGAAGATCTTCAACTCATTGAACTCTTTGGAACTCAAGTCTCTTGAAGAGCCTATCTAC

Chiifu-401 TGGAAGATCTTCAACTCATTGAACTCTTTGGAACTGAAGTCGCTTGAAGAGCCTATCTAC

*********************************** ***** ******** *********

14-490 CTTTTTGGACAGTTCTTTCAGAAGCCCCTTGAATGCTTGACACTCGCTTATTACTTGCCT

12-9 CTTTTTGGACAGTTCTTTCAGAAGCCCCTTGAATGCTTGACACTCGCTTATTACTTGCCT

A21530 CTTTTTGGACAGTTCTTTCAGAAGCCCCTTGAATGCTTGACACTCGCTTATTACTTGCCT

14-401 CTTTTTGGACAGTTCTTTCAGAAGCCGCTTGAATGCTTGGCACTCGCTTATTACTTGCCT

91-112 CTTTTTGGACAGTTCTTTCAGAAGCCGCTTGAATGTTTGACACTCGCTTATTACTTGCCT

A21445 CTTTTTGGACAGTTCTTTCAGAAGCCGCTTGAATGTTTGACACTCGCTTATTACTTGCCT

Chiifu-401 CTTTTTGGACAGTTCTTTCAGAAGCCGCTTGAATGCTTGACACTCGCTTATTACTTGCCT

************************** ******** *** ********************

14-490 CAAAATGCTGGGGATATAGCTCGGAAGTACATAAAGGATCCTCAGTTACTGTCTTTCATT

12-9 CAAAATGCTGGGGATATAGCTCGGAAGTACATAAAGGATCCTCAGTTACTGTCTTTCATT

A21530 CAAAATGCTGGGGATATAGCTCGGAAGTACATAAAGGATCCTCAGTTACTGTCTTTCATT

14-401 CAAAATGCTGGGGATATAGCTCGGAAGTACATAAAGGATCCTCAGTTACTGTCTTTCATT

91-112 CAAAATGCTGGGGACATAGCTCGGAAGTACATAAAGGATCCTCAGTTACTGTCTTTCATT

A21445 CAAAATGCTGGGGACATAGCTCGGAAGTACATAAAGGATCCTCAGTTACTGTCTTTCATT

Chiifu-401 CAAAATGCTGGGGATATAGCTCGGAAGTACATAAAGGATCCTCAGTTACTGTCTTTCATT

************** *********************************************

14-490 GACGCAGAGTGTTTCATTGTGAGCACAGTGAATGCTTTGCAGACGCCGATGATCAATGCA

12-9 GACGCAGAGTGTTTCATTGTGAGCACAGTGAATGCTTTGCAGACGCCGATGATCAATGCA

A21530 GACGCAGAGTGTTTCATTGTGAGTACAGTCAATGCTTTGCAGACGCCGATGATCAATGCA

14-401 GACGCAGAGTGTTTCATTGTGAGCACAGTGAATGCTTTGCAGACGCCGATGATCAATGCA

91-112 GACGCAGAGTGTTTCATTGTGAGCACAGTGAATGCTTTGCAGACGCCAATGATCAATGCA

A21445 GACGCAGAGTGTTTCATTGTGAGTACAGTCAATGCTTTGCAGACGCCAATGATCAATGCA

Chiifu-401 GACGCAGAGTGTTTCATTGTGAGTACAGTCAATGCTTTGCAGACGCCAATGATCAATGCA

*********************** ***** ***************** ************

14-490 AGTATGGTTTTATGTGACAGGCACTATGGAGGGATTAACTACCCTGTTGGTGGTGTTGGT

12-9 AGTATGGTTTTATGTGACAGGCACTATGGAGGGATTAACTACCCTGTTGGTGGTGTTGGT

A21530 AGTATGGTTTTATGTGACAGGCACTATGGAGGGATTAACTACCCTGTTGGTGGTGTTGGT

14-401 AGTATGGTTTTATGTGACAGGCACTATGGAGGGATTAACTACCCTGTTGGTGGTGTTGGT

91-112 AGTATGGTTTTATGTGACAGGCACTATGGAGGGATTAACTACCCTGTTGGTGGTGTTGGT

A21445 AGTATGGTTTTATGTGACAGGCACTATGGAGGGATTAACTACCCTGTTGGTGGTGTTGGT

Chiifu-401 AGTATGGTTTTATGTGACAGGCACTATGGAGGGATTAACTACCCTGTTGGTGGTGTTGGT

************************************************************

14-490 GGGATTGCAAGGTCTTTAGCAGGAGGACTAGTTGATCAAGGAAGTGAAATATTCTACAAA

12-9 GGGATTGCAAGGTCTTTAGCAGGAGGACTAGTTGATCAAGGAAGTGAAATATTCTACAAA

A21530 GGGATTGCAAGGTCTTTAGCAGGAGGACTAGTTGATCAAGGAAGTGAAATATTCTACAAA

14-401 GGGATTGCAAGGTCTTTAGCAGGAGGACTAGTTGATCAAGGCAGTGAAATACTCTACAAA

91-112 GGGATTGCAAGGTCTTTAGCAGGAGGACTAGTTGATCAAGGAAGTGAAATACTCTACAAA

A21445 GGGATTGCAAGGTCTTTAGCAGGAGGACTAGTTGATCAAGGAAGTGAAATACTCTACAAA

Chiifu-401 GGGATTGCAAGGTCTTTAGCAGGAGGACTAGTTGATCAAGGAAGTGAAATACTCTACAAA

***************************************** ********* ********

14-490 GCTAATGTGAAAAGCATAATTCTTGATGATGGAAAGGCTGTGGGTGTAAGGCTAGCAGAT

12-9 GCTAATGTGAAAAGCATAATTCTTGATGATGGAAAGGCTGTGGGTGTAAGGCTAGCAGAT

A21530 GCTAATGTGAAAAGCATAATTCTTGATGATGGAAAGGCTGTGGGTGTAAGGCTAGCAGAT

14-401 GCTAATGTCAAAAGCATAATACTTGATGATGGAAAGGCTGTGGGTGTAAGGCTAGCAGAT

91-112 GCTAATGTGAAAAGCATAATTCTTGATGATGGAAAGGCTGTGGGTGTAAGGCTAGCAGAT

A21445 GCTAATGTGAAAAGCATAATTCTTGATGATGGAAAGGCTGTGGGTGTAAGGCTAGCAGAT

Chiifu-401 GCTAATGTGAAAAGCATAATTCTTGATGATGGAAAGGCTGTGGGTGTAAGGCTAGCAGAT

******** *********** ***************************************

14-490 GGAAGAGAATTCTTCGCTAAAACGATAATTTCTAATGCTACAAGATGGGATACGTTTGGG

12-9 GGAAGAGAATTCTTCGCTAAAACGATAATTTCTAATGCTACAAGATGGGATACGTTTGGG

A21530 GGAAGAGAGTTCTTCGCTAAAACAATAATTTCTAATGCTACAAGATGGGATACGTTTGGG

14-401 GGAAGAGAGTTCTTCGCTAAAACAATAATTTCTAATGCTACAAGATGGGATACGTTTGGG

91-112 GGAAGAGAATTCTTCGCTAAAACGATAATTTCTAATGCTACAAGATGGGATACGTTTGGG

A21445 GGAAGAGAGTTCTTCGCTAAAACAATAATTTCTAATGCTACAAGATGGGATACGTTTGGG

Chiifu-401 GGAAGAGAGTTCTTCGCTAAAACAATAATTTCTAATGCTACAAGATGGGATACGTTTGGG

******** ************** ************************************

14-490 AAGCTGTTGAAAGGAGAAAAGCTTCCAAAAGAAGAAGAAAACTTCCAGAAAGTCTACGTG

12-9 AAGCTGTTGAAAGGAGAAAAGCTTCCAAAAGAAGAAGAAAACTTCCAGAAAGTCTACGTG

A21530 AAGCTGTTGAAAGGAGAAAAGCTTCCAAAAGAAGAAGAAAACTTCCAGAAAGTCTATGTG

14-401 AAGCTGTTGAAAGGAGAAAAGCTTCCAAAGGAAGAAGAAAACTTCCAGAAAGTCTATGTG

91-112 AAGCTGTTGAAAGGAGAAAAGCTTCCAAAAGAAGAAGAAAACTTCCAGAAAGTCTACGTG

A21445 AAGCTGTTGAAAGGAGAAAAGCTTCCAAAAGAAGAAGAAAACTTCCAGAAAGTCTATGTG

Chiifu-401 AAGCTGTTGAAAGGAGAAAAGCTTCCAAAAGAAGAAGAAAACTTCCAGAAAGTCTATGTG

***************************** ************************** ***

14-490 AAGGCTCCATCGTTTCTTTCAATCCACATGGGTGTTAAAGCAGAGGTTCTCCCTCCAGAT

12-9 AAGGCTCCATCGTTTCTTTCAATCCACATGGGTGTTAAAGCAGAGGTTCTCCCTCCAGAT

A21530 AAGGCTCCATCGTTTCTCTCAATCCACATGGGTGTTAAAGCAGAGGTTCTCCCTCCAGAT

14-401 AAGGCTCCATCGTTTCTTTCAATCCACATGGGTGTTAAAGCAGAGGTTCTCCCTCCAGAT

91-112 AAGGCTCCATCGTTTCTTTCAATCCACATGGGTGTTAAAGCAGAGGTTCTCCCTCCAGAT

A21445 AAGGCTCCATCGTTTCTCTCAATCCACATGGGTGTTAAAGCAGAGGTTCTCCCTCCAGAT

Chiifu-401 AAGGCTCCATCGTTTCTCTCAATCCACATGGGTGTTAAAGCAGAGGTTCTCCCTCCAGAT

***************** ******************************************

14-490 ACAGATTGCCATCATTTCGTACTTGAGGATGATTGGAAGAATCTGGAGGAGCCTTATGGC

12-9 ACAGATTGCCATCATTTCGTACTTGAGGATGATTGGAAGAATCTGGAGGAGCCTTATGGC

A21530 ACAGATTGCCATCATTTCGTACTTGAGGATGATTGGAAGAATCTGGAGGAGCCTTATGGC

14-401 ACAGATTGCCATCATTTCGTACTTGAGGATGATTGGAAGAATCTGGAGGAGCCTTATGGC

91-112 ACAGATTGCCATCATTTCGTACTTGAGGATGATTGGAAGAATCTGGAGGAGCCTTATGGC

A21445 ACAGATTGCCATCATTTCGTACTTGAGGATGATTGGAAGAATCTGGAGGAGCCTTATGGC

Chiifu-401 ACAGATTGCCATCATTTCGTACTTGAGGATGATTGGAAGAATCTGGAGGAGCCTTATGGC

************************************************************

14-490 AGTATCTTCCTCAGCATCCCAACCATTCTTGATCCATCCTTGGCTCCAGATGGTCGACAT

12-9 AGTATCTTCCTCAGCATCCCAACCATTCTTGATCCATCCTTGGCTCCAGATGGTCGACAT

A21530 AGTATCTTCCTCAGCATCCCAACCATTCTTGATCCATCCTTGGCTCCAGATGGTCGACAT

14-401 AGTATCTTCCTCAGCATTCCAACCATTCTTGATCCATCTTTGGCTCCAGATGGTCGACAT

91-112 AGTATCTTCCTCAGCATCCCAACCATTCTTGATCCATCCTTGGCTCCAGATGGTCGACAT

A21445 AGTATCTTCCTCAGCATCCCAACCATTCTTGATCCATCCTTGGCTCCAGATGGTCGACAT

Chiifu-401 AGTATCTTCCTCAGCATCCCAACCATTCTTGATCCATCCTTGGCTCCAGATGGTCGACAT

***************** ******************** *********************

14-490 ATACTCCACATATTTACAACTTCTTCCATTGAAGATTGGGAGGGACTCACTCCAAAAGAG

12-9 ATACTCCACATATTTACAACTTCTTCCATTGAAGATTGGGAGGGACTCACTCCAAAAGAG

A21530 ATACTCCACATATTTACAACTTCTTCCATTGAAGATTGGGAGGGACTCACTCCAAAAGAG

14-401 ATACTCCACATATTTACAACTTCTTCCATCGAAGATTGGGAGGGACTCACTCCTAAAGAG

91-112 ATACTCCACATATTTACAACTTCTTCCATTGAAGATTGGGAGGGACTCACTCCAAAAGAG

A21445 ATACTCCACATATTTACAACTTCTTCCATTGAAGATTGGGAGGGACTCACTCCAAAAGAG

Chiifu-401 ATACTCCACATATTTACAACTTCTTCCATTGAAGATTGGGAGGGACTCACTCCAAAAGAG

***************************** *********************** ******

14-490 TATGAGGCTAAAAAAGAAGAGGTGGCAGCTGGAATCATACAGAGGCTAGAGAAAAAACTG

12-9 TATGAGGCTAAAAAAGAAGAGGTGGCAGCTGGAATCATACAGAGGCTAGAGAAAAAACTG

A21530 TATGAGGCTAAAAAAGAAGAGGTGGCAGCTGGAATCATACAGAGGCTAGAGAAAAAACTG

14-401 TACGAGGCTAAAAAAGAAGAGGTGGCAGCTGGAATCATACAGAGGCTAGAGAAAAAACTG

91-112 TATGAGGCTAAAAAAGAAGAGGTGGCAGCTGGAATCATACAGAGGCTAGAGAAAAAACTG

A21445 TATGAGGCTAAAAAAGAAGAGGTGGCAGCTGGAATCATACAGAGGCTAGAGAAAAAACTG

Chiifu-401 TATGAGGCTAAAAAAGAAGAGGTGGCAGCTGGAATCATACAGAGGCTAGAGAAAAAACTG

** *********************************************************

14-490 TTTCCTGGGCTCAGTTCATCTATTACTTTTAAGGAGGTGGGCACACCAAGAACACACAGG

12-9 TTTCCTGGGCTCAGTTCATCTATTACTTTTAAGGAGGTGGGCACACCAAGAACACACAGG

A21530 TTTCCTGGGCTCAGTTCATCTATTACTTTTAAGGAGGTGGGCACACCAAGAACACACAGG

14-401 TTTCCTGGTCTCAGTTCATCTATTACTTTTAAGGAGGTGGGCACACCAAGAACACACAGG

91-112 TTTCCTGGTCTCAGTTCATCTATTACTTTTAAGGAGGTGGGCACACCAAGAACACACAGA

A21445 TTTCCTGGTCTCAGTTCATCTATTACTTTTAAGGAGGTGGGCACACCAAGAACACACAGA

Chiifu-401 TTTCCTGGGCTCAGTTCATCTATTACTTTTAAGGAGGTGGGCACACCAAGAACACACAGG

******** **************************************************

14-490 CGATATCTTGCTAGGGATAAGGGAACGTATGGACCAATGCCAAGAGGAACACCAAAAGGT

12-9 CGATATCTTGCTAGGGATAAGGGAACGTATGGACCAATGCCAAGAGGAACACCAAAAGGT

A21530 CGATATCTTGCTAGGGATAAGGGAACGTATGGACCAATGCCAAGAGGAACACCAAAAGGT

14-401 CGATATCTTGCTAGGGATAAGGGAACGTATGGACCAATGCCAAGAGGAACACCAAAAGGT

91-112 CGATATCTTGCTCGGGACAAGGGAACGTATGGACCAATGCCAAGAGGAACACCAAAAGGT

A21445 CGATATCTTGCTCGGGACAAGGGAACGTATGGACCAATGCCAAGAGGAACACCAAAAGGT

Chiifu-401 CGATATCTTGCTAGGGATAAGGGAACGTATGGACCAATGCCAAGAGGAACACCAAAAGGT

************ **** ******************************************

14-490 TTACTAGGCATGCCGTTTAACACAACTGCTATAGATGGTTTGTACTGCGTTGGGGATAGT

12-9 TTACTAGGCATGCCGTTTAACACAACTGCTATAGATGGTTTGTACTGCGTTGGGGATAGT

A21530 TTACTAGGCATGCCGTTTAACACAACTGCTATAGATGGTTTGTACTGCGTTGGGGATAGT

14-401 GTACTAGGCATGCCGTTTAACACAACTGCTATAGATGGTTTGTACTGCGTTGGGGATAGT

91-112 TTACTGGGCATGCCGTTTAACACAACTGCTATAGATGGTTTGTACTGCGTTGGGGATAGT

A21445 TTACTGGGCATGCCGTTTAACACAACTGCTATAGATGGTTTGTACTGCGTTGGGGATAGT

Chiifu-401 TTACTAGGCATGCCGTTTAACACAACTGCTATAGATGGTTTGTACTGCGTTGGGGATAGT

**** ******************************************************

14-490 TGTTTTCCTGGTCAGGGAGTTATAGCTGTGGCTTTCTCAGGAGTGATGTGTGCTCATCGT

12-9 TGTTTTCCTGGTCAGGGAGTTATAGCTGTGGCTTTCTCAGGAGTGATGTGTGCTCATCGT

A21530 TGTTTTCCTGGTCAGGGAGTTATAGCTGTGGCTTTCTCAGGAGTGATGTGTGCTCATCGT

14-401 TGTTTTCCTGGTCAGGGAGTTATAGCTGTAGCTTTCTCAGGAGTGATGTGTGCTCATCGT

91-112 TGTTTTCCTGGTCAGGGAGTTATAGCTGTGGCTTTCTCAGGAGTGATGTGTGCTCATCGT

A21445 TGTTTTCCTGGTCAGGGAGTTATAGCTGTGGCTTTCTCAGGAGTGATGTGTGCTCATCGT

Chiifu-401 TGTTTTCCTGGTCAGGGAGTTATAGCTGTGGCTTTCTCAGGAGTGATGTGTGCTCATCGT

***************************** ******************************

14-490 GTAGCTGCTGACATTGT-TTTGGCAGGAAACCTCGCTTTTGCGGTTTTGGCGGAAAAACT

12-9 GTAGCTGCTGACATTGT-TTTGGCAGGAAACCTCGCTTTTGCGGTTTTGGCGGAAAAACT

A21530 GTAGCTGCTGACATTGGGCTTGAGAGAAAATCAAAGGTACTTGATGCTGGTC------TT

14-401 GTAGCTGCTGACATTGGGCTTGAGAGAAAATCAAAGGTACTTGATGCTGGCC------TT

91-112 GTAGCTGCTGACATTGGGCTTGAGAGAAAATCAAAGGTACTTGATGCTGGTC------TT

A21445 GTAGCTGCTGACATTGGGCTTGAGAGAAAATCAAAGGTACTTGATGCTGGTC------TT

Chiifu-401 GTAGCTGCTGACATTGGGCTTGAGAGAAAATCAAAGGTACTTGATGCTGGTC------TT

**************** *** ** *** * * * * *** *

14-490 CGTTTTTGATTTTGACGGAAAAACTTGTTTTTACGGTTTTGGGGAAACTCGGTTTTCGGC

12-9 CGTTTTTGATTTTGACGGAAAAACTTGTTTTTACGGTTTTGGGGAAACTCGGTTTTCGGC

A21530 CTTGGTTTACTTGGTTGGTTAAGGACACTCGCATAG------------------------

14-401 CTTGGTTTACTTGGTTGGTTAAGGACACTCGCATAG------------------------

91-112 CTTGGTTTACTTGGTTGGTTAAGGACACTCGCATAG------------------------

A21445 CTTGGTTTACTTGGTTGGTTAAGGACACTCGCATAG------------------------

Chiifu-401 CTTGGTTTACTTGGTTGGTTAAGGACACTCGCATAGG-----------------------

* * ** * ** * ** ** * * *

14-490 TTTGACGGGAAAACTCGATTTTTCGATTTTGGCGGGAAAACTCGATTTTGCGGTTTTGGC

12-9 TTTGACGGGAAAACTCGATTTTTCGATTTTGGCGGGAAAACTCGATTTTGCGGTTTTGGC

A21530 ------------------------------------------------------------

14-401 ------------------------------------------------------------

91-112 ------------------------------------------------------------

A21445 ------------------------------------------------------------

Chiifu-401 ------------------------------------------------------------

14-490 GGGAAAACTCGGTTTTTCTGTTTTGGCGGAAAAACCATGTTTTTCGCTTTCGGCAGTAAA

12-9 GGGAAAACTCGGTTTTTCTGTTTTGGCGGAAAAACCATGTTTTTCGCTTTCGGCAGTAAA

A21530 ------------------------------------------------------------

14-401 ------------------------------------------------------------

91-112 ------------------------------------------------------------

A21445 ------------------------------------------------------------

Chiifu-401 ------------------------------------------------------------

14-490 AATCGTTTTGGCGGGAAAATTGAGTTTTACGGCATTGGCGGGAAAACACCTTTTGCGGTT

12-9 AATCGTTTTGGCGGGAAAATTGAGTTTTACGGCATTGGCGGGAAAACACCTTTTGCGGTT

A21530 ------------------------------------------------------------

14-401 ------------------------------------------------------------

91-112 ------------------------------------------------------------

A21445 ------------------------------------------------------------

Chiifu-401 ------------------------------------------------------------

14-490 TTGGCGGAAAAACTCGATTTTGGGGCTTTCAGTCGGAAAACTCGATTTTACGGTTTTAGC

12-9 TTGGCGGAAAAACTCGATTTTGGGGCTTTCAGTCGGAAAACTCGATTTTACGGTTTTAGC

A21530 ------------------------------------------------------------

14-401 ------------------------------------------------------------

91-112 ------------------------------------------------------------

A21445 ------------------------------------------------------------

Chiifu-401 ------------------------------------------------------------

14-490 GGGAAAACTCAGTTTTGCAGTTTTGGTGAGAAAACTCAGTTTTGCGGTTTTGGCGGGAAA

12-9 GGGAAAACTCAGTTTTGCAGTTTTGGTGAGAAAACTCAGTTTTGCGGTTTTGGCGGGAAA

A21530 ------------------------------------------------------------

14-401 ------------------------------------------------------------

91-112 ------------------------------------------------------------

A21445 ------------------------------------------------------------

Chiifu-401 ------------------------------------------------------------

14-490 CTTAGTTTTATGGTTTTGGCGGAAAAACAAGTTTTGTGGTTTCGGTAGAAAACCTCGATT

12-9 CTTAGTTTTATGGTTTTGGCGGAAAAACAAGTTTTGTGGTTTCGGTAGAAAACCTCGATT

A21530 ------------------------------------------------------------

14-401 ------------------------------------------------------------

91-112 ------------------------------------------------------------

A21445 ------------------------------------------------------------

Chiifu-401 ------------------------------------------------------------

14-490 TTTCGGTTTCGGCGGGAAAACTCGTTTTTGGTTTCTGTGA

12-9 TTTCGGTTTCGGCGGGAAAACTCGTTTTTGGTTTCTGTGA

A21530 ----------------------------------------

14-401 ----------------------------------------

91-112 ----------------------------------------

A21445 ----------------------------------------

Chiifu-401 ----------------------------------------
