# Supplementary material for: Transcriptome Analysis of Orange Head Chinese Cabbage (Brassica rapa L. ssp. pekinensis) and Molecular Marker Development
Source: Int J Genomics. 2017 Apr 2;2017:6835810. doi: 10.1155/2017/6835810 (PMC5392394; doi:10.1155/2017/6835810)

Fig. S1 Validation of the SNP (C^952^ to T^952^) in different white and orange cultivars and F_2_ populations. A: Validation of the SNP (C^952^ to T^952^) in the parents 14-401 and 14-490, and its F_2_ individuals. Among the F_2_ individuals, 1-11 are the lines with the white inner leaves, and 12-19 are the lines with the orange inner leaves. B: Validation of the SNP (C^952^ to T^952^) in the breeding lines. 1-11 are 663, 1466, 1469, 1492, 1505, 1510, 1720, Hanxiu, Jindianchunwang, Kaichun and Ribenxiayang with the white or yellow inner leaves. 12-23 are 1480, 14-102, 14-245,14-253, 14-257, 14-277, 14-426, 14-662, 14-669, Changyanjubao, Shenmengjuhongxin and Shenshijuhongxin with the orange inner leaves.


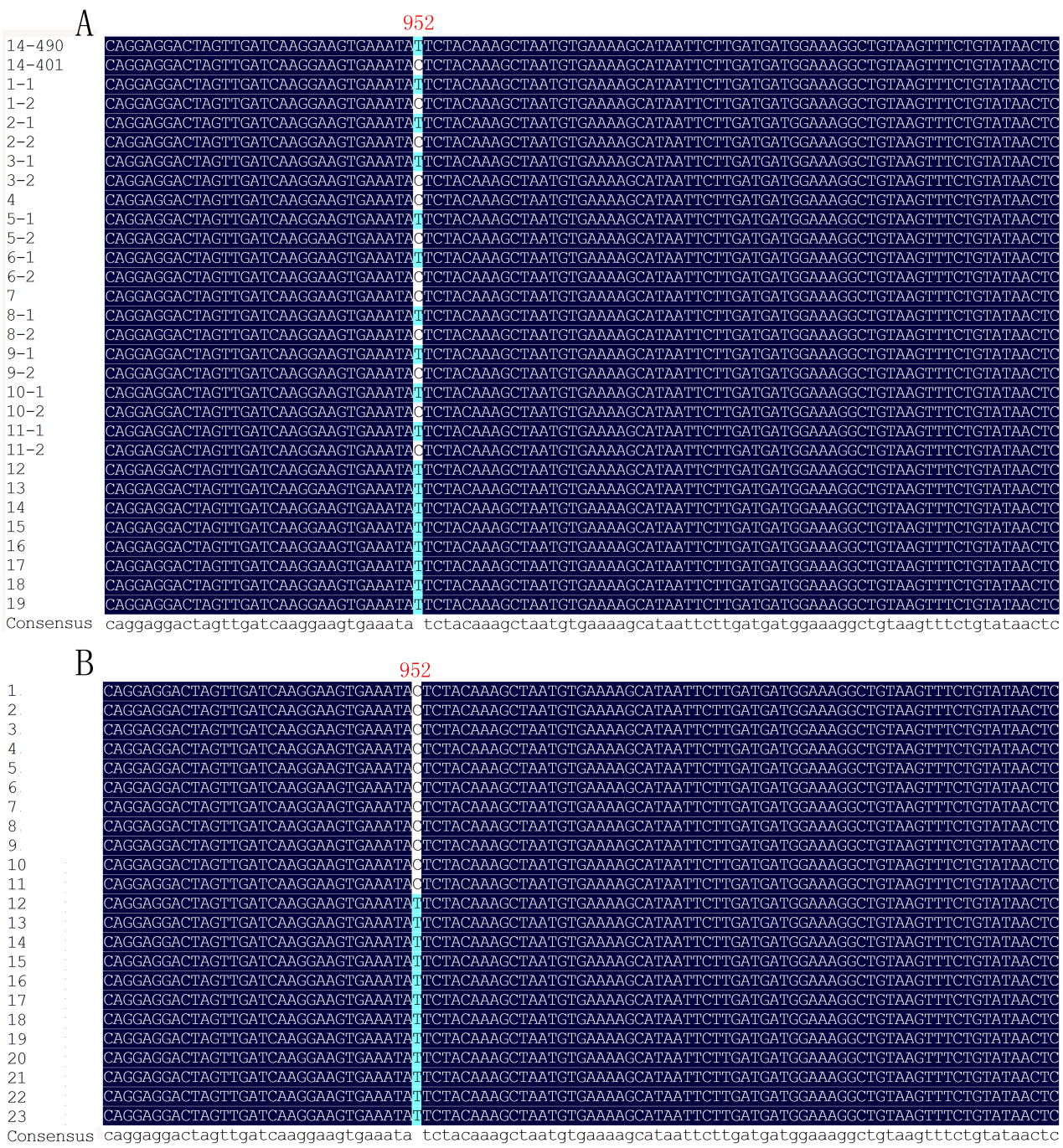

Supplement: Supplementary file 4 [file 6835810.f4.docx]
