# Supplementary material for: Transcriptome Analysis of Orange Head Chinese Cabbage (Brassica rapa L. ssp. pekinensis) and Molecular Marker Development
Source: Int J Genomics. 2017 Apr 2;2017:6835810. doi: 10.1155/2017/6835810 (PMC5392394; doi:10.1155/2017/6835810)
Supplement: Supplementary file 6 [file 6835810.f6.docx]

File S3 Optimal alignment of the promoter sequences of Bra031539 from 14-401, 14-490, 12-9 and 91-112 (Su et al, 2014).

14-401 ----------GGTACCGGGGACCTCTAGAGATT-----TTTTCTTGATGGAGTGAAAGGA

14-490 --------------------------------------TTTTCTTGATGGAGTGAAAGGA

91-112 CACTCCTTTCTCTGACGATAGTCTCATAACCTTGTCCATTTTCTTGATGGAGTGAAAGGA

12-9 CACTCCTTTCTCTGACGATAGTCTCATAACCTTGTCCATTTTCTTGATGGAGTGAAAGGA

**********************

14-401 GTCTTATAACAAAGACTCCTTGAGACCAAACCCGGAGATCGAGCTTGGTGTTGCTTGTGA

14-490 GTCTTATAACAAAGACTCCTTGAGACCAAACCCGGAGATCGAGCTTGGTGTTGCTTGTGA

91-112 GTCTTATAACAAAGACTCCTTGAGACCAAACCCGGAGATCGAGCTTGGTGTTGCTTGTGA

12-9 GTCTTATAACAAAGACTCCTTGAGACCAAACCCGGAGATCGAGCTTGGTGTTGCTTGTGA

************************************************************

14-401 GTCTGGTTACTTGAGTCTCAAGGCTATTTATAACCAAGTAGAAGAAGAATCAGAATCCAA

14-490 GTCTGGTTACTTGAGTCTCAAGGCTATTTATAACCAAGTAGAAGAAGAATCAGAATCCAA

91-112 GTCTGGTTACTTGAGTCTCAAGGCTATTTATAACCAAGTAGAAGAAGAATCAGAATCCAA

12-9 GTCTGGTTACTTGAGTCTCAAGGCTATTTATAACCAAGTAGAAGAAGAATCAGAATCCAA

************************************************************

14-401 ATACTTTTTTTAAAGGAAGAGATCACAAGAAGATCGTGTTGAAAAGGATCCATAAAGAGG

14-490 ATACTTTTTTTAAAGGAAGAGATCACAAGAAGATCGTGTTGAAAAGGATCCATAAAGAGG

91-112 ATACTTTTTTTAAAGGAAGAGATCACAAGAAGATCGTGTTGAAAAGGATCCATAAAGAGG

12-9 ATACTTTTTTTAAAGGAAGAGATCACAAGAAGATCGTGTTGAAAAGGATCCATAAAGAGG

************************************************************

14-401 CTTTACTATGAACAAGATGATTCGAGGAGACCGAACCTTTTCTTAAGAGTTAGCCGCGTT

14-490 CTTTACTATGAACAAGATGATTCGAGGAGACCGAACCTTTTCTTAAGAGTTAGCCGCGTT

91-112 CTTTACTATGAACAAGATGATTCGAGGAGACCGAACCTTTTCTTAAGAGTTAGCCGCGTT

12-9 CTTTACTATGAACAAGATGATTCGAGGAGACCGAACCTTTTCTTAAGAGTTAGCCGCGTT

************************************************************

14-401 ACACACCTCTTATATATTTTTCTTGAAAGAACCATGAAGAGTTGCATTGGCTCATTCCAT

14-490 ACACACCTCTTA----TTTTTCTTGAAAGAACCATGAAGAGTTGCATTGGCTCATTCCAT

91-112 ACACACCTCTTA----TTTTTCTTGAAAGAACCATGAAGAGTTGCATTGGCTCATTCCAT

12-9 ACACACCTCTTA----TTTTTCTTGAAAGAACCATGAAGAGTTGCATTGGCTCATTCCAT

************ ********************************************

14-401 GTTCTTATATCCATAGATTCAAAGATAAGTTCTCAACGATACTCTTACAAGTAGCTAAAA

14-490 GTTCTTATATCCATAGATTCAAAGATAAGTTCTCAACGATACTCTTACAAGTAGCTAAAA

91-112 GTTCTTATATCCATAGATTCAAAGATAAGTTCTCAACGATACTCTTACAAGTAGCTAAAA

12-9 GTTCTTATATCCATAGATTCAAAGATAAGTTCTCAACGATACTCTTACAAGTAGCTAAAA

************************************************************

14-401 AAGAGAGCAAGATGATTCGAGGAGACCGAACCTTTTCTTGGGCGTTAGCCGCGTTACACA

14-490 AAGAGAGCAAGATGATTCGAGGAGACCGAACCTTTTCTTGGGCGTTAGCCGCGTTACACA

91-112 AAGAGAGCAAGATGATTCGAGGA--CCGAACCTTTTCTTGGGCGTTAGCCGCGTTACACA

12-9 AAGAGAGCAAGATGATTCGAGGAGACCGAACCTTTTCTTGGGCGTTAGCCGCGTTACACA

*********************** ***********************************

14-401 CCTCTTGTTTTTCTTGAAAGAACCAAGAAGAGTTGCATTGGTCATTCCAACGTTTTATTT

14-490 CCTCTTGTTTTTCTTGAAAGAACCAAGAAGAGTTGCATTGGTCATTCCAACGTTTTATTT

91-112 CCTCTTGTTTTTCTTGAAAGAACCAAGAAGAGTTGCATTGGTCATTCCAACGTTTTATTT

12-9 CCTCTTGTTTTTCTTGAAAGAACCAAGAAGAGTTGCATTGGTCATTCCAACGTTTTATTT

************************************************************

14-401 CATAGATTCAAAGATACGGTCTCAACATACTCGTTCAAGTTGCTAAGAAGACAGGAATAG

14-490 CATAGATTCAAAGATACGGTCTCAACATACTCGTTCAAGTTGCTAAGAAGACAGGAATAG

91-112 CATAGATTCAAAGATACGGTCTCAACATACTCGTTCAAGTTGCTAAGAAGACAGGAATAG

12-9 CATAGATTCAAAGATACGGTCTCAACATACTCGTTCAAGTTGCTAAGAAGACAGGAATAG

************************************************************

14-401 TCGTAGAATATTAGTTGATGCTTGATGCTT-------TTGGGCCATTTGATTTATTCTGT

14-490 TCGTAGAATATTAGTTGATGCTTGATGCTTGATGCTTTTGGGCCATTTGATTTATTCTGT

91-112 TCGTAGAATATTAGTTGATGCTTGATGCTTGATGCTTTTGGGCCATTTGATTTATTCTGT

12-9 TCGTAGAATATTAGTTGATGCTTGATGCTTGATGCTTTTGGGCCATTTGATTTATTCTGT

****************************** ***********************

14-401 AGATTCTATGAATACGGGACATGGAATCAGATTCACCACGAAATTAAACCTTTCTGTCCT

14-490 AGATTCTATGAATACGGGACATGGAATCAGATTCACCACGAAATTAAACCTTTCTGTCCT

91-112 AGATTCTATGAATACGGGACATGGAATCAGATTCACCACGAAATTAAACCTTTCTGTCCT

12-9 AGATTCTATGAATACGGGACATGGAATCAGATTCACCACGAAATTAAACCTTTCTGTCCT

************************************************************

14-401 TTTTCGCGCCATATAAATCCTATGAGAGTTTTAAAAAAACCATGATACAAAAAAGATTAG

14-490 TTT-CGCGCCATATAAATCCTATGAGAGTTT-AAAAAAAGCATGATACAAAAAAGATTAG

91-112 TTT-CGCGCCATATAAATCCTACGAGAGTTT-AAACAAAACATGATACAAAAAAGATTAG

12-9 TTT-CGCGCCATATAAATCCTATGAGAGTTT-AAAAAAAGCATGATACAAAAAAGATTAG

*** ****************** ******** *** *** ********************

14-401 CATTATTAATACCTATAATAATCTAATGAGTATTGGCGATGTTCTAAAAATCGTTAGGCA

14-490 CTTTATTAATACCTATAATAATCTAATGTGTATTGGCAATGTTCTAAAAATCGCTAGACA

91-112 CATTATTAATACCTATAATAATCTAATGAGCATTGGCAATGTCCTAAAAATCGTTAGGCA

12-9 CTTTATTAATACCTATAATAATCTAATGTGTATTGGCAATGTTCTAAAAATCGCTAGACA

* ************************** * ****** **** ********** *** **

14-401 GAGTAGTCAACCGTTTTATAGAAGATTATTGTTTACGCGGTTGCTTACGCTGATATTTTA

14-490 ------------------------------------------------------------

91-112 G--TAGTCAACCGTCTTATAGAAGATTATTGTTTACGCGGTTGCTTACGCTGATATTTTA

12-9 C--TAATCTAAC------------------------------------------------

14-401 GAATTTTTAGACCGAATTTTTTAAAATCAATTTGAAAAAATCGTTTCGCTCATATCCGAT

14-490 --------------------------CTAATCT----AACTCATTTTTTTCATATCCGAT

91-112 GAATTTTTAGACCAAATTTTTTAAAATCAATTTGAAAAAATCGTTTCGCTCATATTCGAT

12-9 ----------------------------------------TCATTTTTTTCATATCCGAT

** *** ****** ****

14-401 TTGCCGATTAGGTGCCATCTATACCGATTTTTAAAACAT---TAGGTATTATTTAATGTC

14-490 TTACCGATTAGGTGCTATCTATGCCGATTTTTAAATCATCATTAGGTATTATTTAATGTC

91-112 TTGCCGATTAGGTGCCATTTATACCGATTTTTAAAACA---TTAGGTATTATTTAATGTC

12-9 TTACCGATTAGGTGCTATCTATGCCGATTTTTAAATCATCATTAGGTATTATTTAATGTC

** ************ ** *** ************ ** ******************

14-401 AGTTTTTTTTTTTTTTGTCATTGATAATTTATTAAACAAAGGCTACAGGAA-GGCCCAAC

14-490 AGTTTTTTTTT---TTGTCATTGATAATTTATTAAACAAAGGCTACAGGAA-GGCCCAAC

91-112 AATTTTTTTTTT--TTGTCATTGATAATTTATTAAACAAAGGCTACAGGAAAGGCCCAAC

12-9 AGTTTTTTTTTT--T-GTCATTGATAATTTATTAAACAAAGGCTACAGGAA-GGCCCAAC

* ********* * *********************************** ********

14-401 GGATAGGTGTAACAAACAAAGAGACAAAGGCCCGAAAGGTCCAACGACAATAAACTAAAA

14-490 GGATAGGTGTAACAAACAAAGAGACAAAGGCCGAAAAGGCCCAACAACAACAAACTAAAA

91-112 GGATAGGTGTAACAAACAAAGAGACAAAGGCCCGAAAGGTCCAACGACAATAAACTAAAA

12-9 GGATAGGTGTAACAAACAAAGAGACAAAGGCCGAAAAGGCCCAACAACAACAAACTAAAA

******************************** ***** ***** **** *********

14-401 GACATTACGCCCCAAAATTAATGAATAATCAACGGCCACGATTAGAAGTCACTAAAATAA

14-490 GACATTACGGCCCAAAATTAATGAATAATCAACGGCCACGATTAGAAGTCACTAAAATAA

91-112 GACATTACGCCCCAAAATTAATGAATAATCACGGGCCACGATTAGA-GTCACTAAAATAA

12-9 GACATTACGGCCCAAAATTAATGAATAATCAACGGCCACGATTAGAAGTCACTAAAATAA

********* ********************* ************* *************

14-401 AGTTTTTTTTTTTTTTTTTT-TGTAATATCCTGCACTTCTGAGTGTTCTTCACCAAACAT

14-490 AGTTTTTTTTTTTTTTTTTTCTGTAATATCCTGCACTTCCGAGTGTTCTTCACCAAACAT

91-112 AGTTTTTTTTTTTTG---------------------------------------------

12-9 AGTTTTTTTTTTTTT---------------------------------------------

**************
